# NLRP3 inflammasome activation by mitochondrial ROS in bronchial epithelial cells is required for allergic inflammation

**DOI:** 10.1038/cddis.2014.460

**Published:** 2014-10-30

**Authors:** S R Kim, D I Kim, S H Kim, H Lee, K S Lee, S H Cho, Y C Lee

**Affiliations:** 1 Department of Internal Medicine, Research Center for Pulmonary Disorders, Chonbuk National University Medical School, Research Institute of Clinical Medicine of Chonbuk National University – Biomedical Research Institute of Chonbuk National University Hospital, Deokjin-gu, Jeonju, South Korea; 2 Department of Product Strategy and Development, LG Life Sciences Ltd, Seoul, Korea; 3 Division of Allergy-Immunology, Department of Medicine, Northwestern University, Feinberg School of Medicine, Chicago, IL, USA

## Abstract

Abnormality in mitochondria has been suggested to be associated with development of allergic airway disorders. In this study, to evaluate the relationship between mitochondrial reactive oxygen species (ROS) and NLRP3 inflammasome activation in allergic asthma, we used a newly developed mitochondrial ROS inhibitor, NecroX-5. NecroX-5 reduced the increase of mitochondrial ROS generation in airway inflammatory cells, as well as bronchial epithelial cells, NLRP3 inflammasome activation, the nuclear translocation of nuclear factor-*κ*B, increased expression of various inflammatory mediators and pathophysiological features of allergic asthma in mice. Finally, blockade of IL-1*β* substantially reduced airway inflammation and hyperresponsiveness in the asthmatic mice. These findings suggest that mitochondrial ROS have a critical role in the pathogenesis of allergic airway inflammation through the modulation of NLRP3 inflammasome activation, providing a novel role of airway epithelial cells expressing NLRP3 inflammasome as an immune responder.

There is now substantial evidence that an excess of reactive oxygen species (ROS) has an important role in the pathogenesis of airway inflammation and tissue injury observed in asthma, which consists of epithelial cell damage, cell shedding and airway hyperresponsiveness.^1, 2, 3^ In addition, increased oxidative stress is related to severity of asthma, propagation of inflammatory response and reduction of responsiveness to corticosteroids.^4^ Thus, considerable research efforts have been focused on understanding the mechanism of oxidative stress-mediated airway inflammation and finding better antioxidants.

Mitochondria and the Nox family of nicotinamide adenine dinucleotide phosphate (NADPH) oxidase are the two major sources of ROS that are induced by external stimuli, and the mitochondria respiratory chain is considered an important site of ROS production within most cells.^5^ Mitochondria are dynamic double membrane organelles and possess their own genome and proteome.^6^ They are associated with the synthesis and catabolism of metabolites, generation and detoxification of ROS, apoptosis, regulation of cytoplasmic and mitochondrial matrix calcium and generation of adenosine triphosphate by oxidative phosphorylation.^7^ Recently, apart from these classical functions of mitochondria, a new and exciting role for mitochondria has been revealed in various inflammatory disorders such as infectious diseases, neurodegenerative diseases, cerebrovascular diseases and metabolic diseases,^7, 8, 9, 10^ especially in the activation and control of innate immune responses. Moreover, recent studies have suggested that abnormality in mitochondria is associated with development of asthma.^11,12^ However, the precise role of the excess of mitochondrial ROS generation in the development of allergic airway inflammation is not well understood.

Cellular stress or tissue damage is recognized by the pattern recognition receptors (PRRs) of the innate immune system. The allergens such as dust mite and molds contain protease activity and/or innate PRR ligands for the Toll-like receptor (TLR), C-type lectin receptor, and/or nucleotide-binding domain, leucine-rich repeat-containing protein (NLR) families that facilitate their immunogenicity. Among them, several members of the cytosolic NLR family (NLRP1, NLRP3 and NLRC4) act as central components of the multiprotein inflammasome complex.^13^ A number of studies have shown that NLRP3 inflammasome, which consists of NLRP3, apoptosis-associated speck-like protein containing a carboxy-terminal CARD (ASC), and caspase-1 is related to mitochondrial dysfunction.^14^ Activation of NRLP3 inflammasome can be induced by intracellular ROS generation in response to a variety of cellular stress.^15, 16, 17^ More interestingly, studies have demonstrated that NLRP3 inflammasome activation is critical for the induction of allergic airway inflammation in bronchial asthma,^18,19^ with increased understanding of how adaptive and innate immunity generate downstream pathology of allergic inflammation.^20^ In fact, although bronchial asthma has been characterized by reversible airway obstruction, airway hyperresponsiveness, infiltration of eosinophils and CD4+ T helper (Th) type 2 cells into the airway submucosa, mucus hypersecretion and airway remodeling, severe and fatal asthma has been reported to be mediated by neutrophils.^21^ Therefore, the several novel therapeutic approaches focused largely on Th2-driven pathways of asthmatic inflammation have not proven successful in treatment for many patients with asthma in clinical practice. For this reason, recent research has preferred the use of allergens such as dust mite, molds and microbial compounds including lipopolysaccharide (LPS), which show the neutrophilic inflammation, to the use of surrogate allergen ovalbumin (OVA).^20^ However, to date, there is little information regarding the relationship between mitochondrial dysfunction, specifically mitochondrial ROS and NLRP3 inflammasome activation, in the pathogenesis of neutrophilic allergic inflammation of bronchial asthma.

Recently, a novel mitochondrial ROS inhibitor, NecroX-5, has been synthesized and developed by LG Life Sciences (Seoul, Korea). It is one of the derivatives of NecroX series compounds, whose chemical composition is C25H31N3O3S.2CH4O3S with molecular weight 453.61.^22^ Moreover, increasing evidence indicates the excellent efficacy of this chemical as a potent and specific mitochondria targeted antioxidant.^23,24^ In this study, we aimed to evaluate the role of mitochondrial ROS in the induction and/or maintenance of neutrophilic, as well as eosinophilic allergic airway inflammation in bronchial asthma, focusing on the relationship between mitochondrial ROS and NLRP3 inflammasome activation.

## Results

### Total cellular ROS and mitochondrial ROS are increased in cells from lung of mice sensitized with OVA and LPS and challenged with OVA (OVA_LPS_-OVA mice)

**Figure 1:**
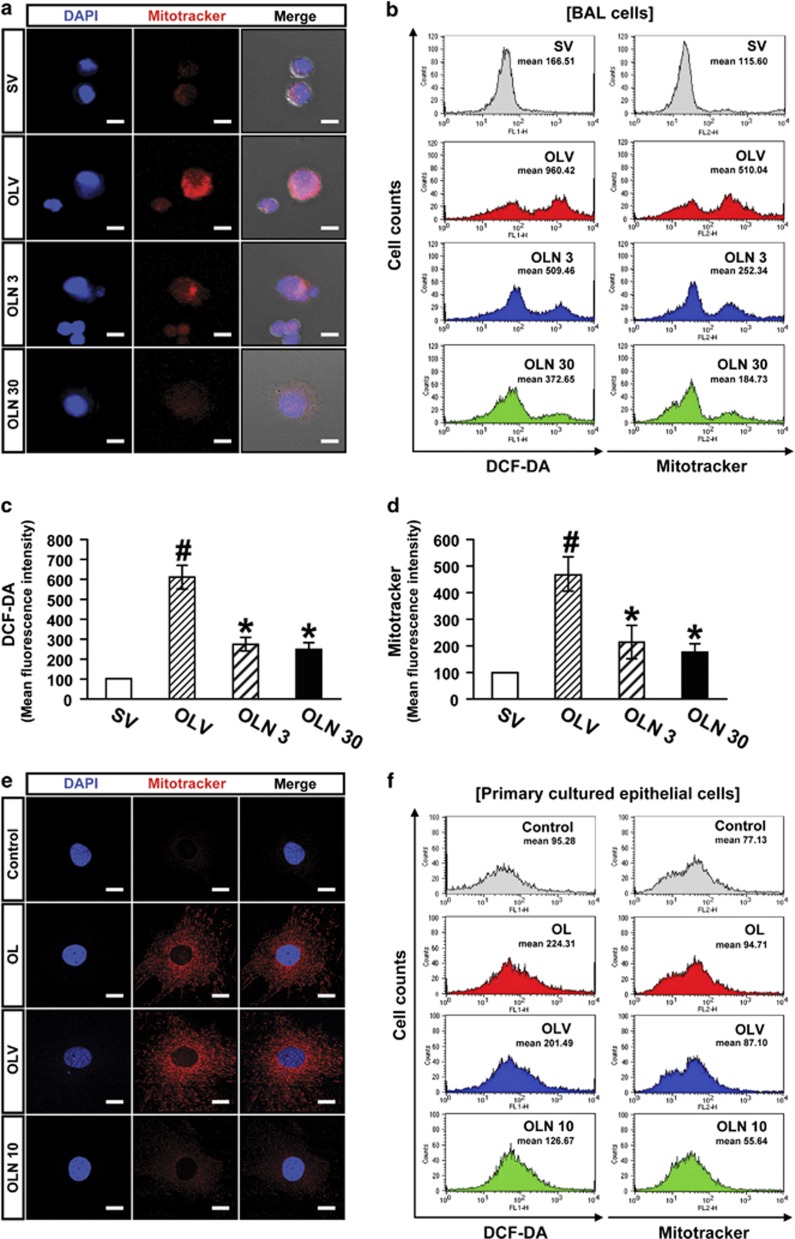
Generation of Intracellular ROS and mitochondrial ROS in BAL cells and primary cultured tracheal epithelial cells from OVA_LPS_-OVA mice. BAL cells were obtained at 48 h after the challenge in SAL-SAL mice administered with drug vehicle (SV), OVA_LPS_-OVA mice administered with drug vehicle (OLV), OVA_LPS_-OVA mice administered with 3 mg/kg Necrox-5 (OLN 3) or OVA_LPS_-OVA mice administered with 30 mg/kg Necrox-5 (OLN 30). The experiment was performed with the primary cultured tracheal epithelial cells from SAL-SAL mice without any treatment (Control), OVA_LPS_-OVA mice with no treatment (OL), or OVA_LPS_-OVA mice at 90 min after the treatment with drug vehicle (OLV) or 10 *μ*M Necrox-5 (OLN 10). Representative confocal laser immunofluorescence photomicrography of BAL cells (**a**) and primary cultured tracheal epithelial cells (**e**) showed the localization of mitochondrial ROS (the center panels, red fluorescence views) in the cells. The blue fluorescent DAPI stain was used for nuclear localization. The left and the right panels presented phase contrast views and the merger views, respectively. Bars indicate scale of 10 *μ*m (**a**) or 20 *μ*m (**e**). Histogram analysis for ROS generation in BAL cells (**b**) and primary cultured tracheal epithelial cells (**f**). (**c**) DCF fluorescence intensity of intracelluar ROS in BAL cells. (**d**) MitoTracker fluorescence intensity of mitochondrial ROS in BAL cells. The ROS levels are presented as the relative ratio of values in experimental groups to those in SV. The relative ratio of ROS levels in the BAL cells of SV arbitrarily presented as 100. Bars represent mean±S.E.M. from seven mice per group. ^*#*^*P*<0.05 *versus* SV or control; *P<0.05 *versus* OLV

Intensity of fluorescence was significantly higher in bronchoalveolar lavage (BAL) cells (Figure 1a) and primary cultured tracheal epithelial cells (Figure 1e) from OVA_LPS_-OVA mice than those from control mice. The enhancement of fluorescence intensity in BAL cells or primary cultured tracheal epithelial cells from OVA_LPS_-OVA mice was inhibited by treatment with NecroX-5. Additional FACScan analysis also showed that mitochondrial ROS generation as well as total cellular ROS generation was significantly upregulated in BAL cells (Figures 1b–d) and primary cultured tracheal epithelial cells (Figure 1f) from OVA_LPS_-OVA mice. The increases of ROS generations in both cells from OVA_LPS_-OVA mice were significantly decreased by treatment with NecroX-5.

### Changes of mitochondrial DNA (mtDNA) in lung tissues of OVA_LPS_-OVA mice

**Figure 2:**
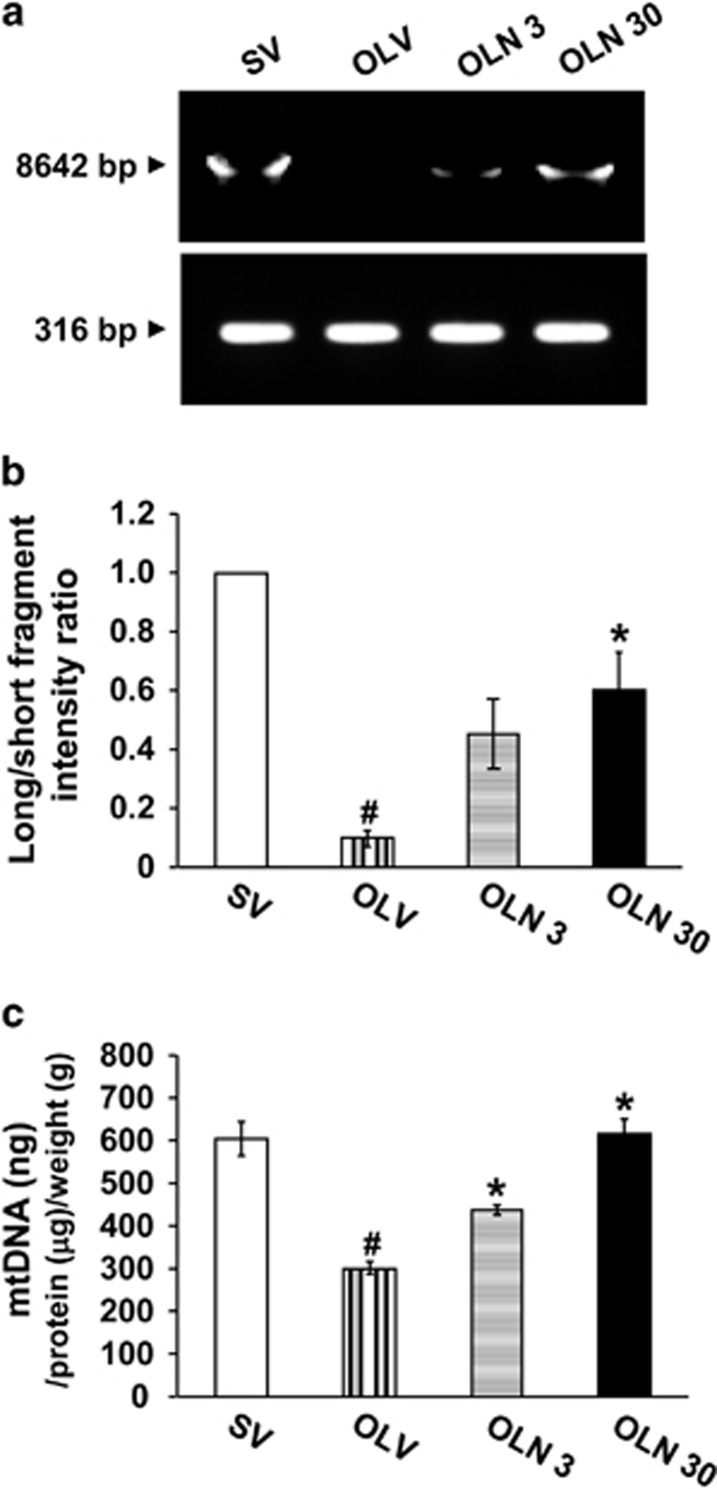
Effects of NecroX-5 on the integrity and content of mtDNA in lung tissues of OVA_LPS_-OVA mice. Sampling was performed at 48 h after the last challenge in SV, OLV, OLN 3 or OLN 30. (**a**) Representative PCR analyses for both a long (8642-bp) mtDNA fragment and a short (316-bp) mtDNA fragment from the lung tissues of OVA_LPS_-OVA mice. (**b**) The long fragment/short mtDNA fragment hybridization ratio in the lung tissues of OVA_LPS_-OVA mice. The ratio of SV is arbitrarily presented as 1. (**c**) Changes of the content of mtDNA in the lung tissues of OVA_LPS_-OVA mice. The levels of mtDNA were presented as ng/1 *μ*g of protein/1 g of lung tissues. Data represent mean±S.E.M. from six mice per group. ^#^*P*<0.05 *versus* SV; *P<0.05 *versus* OLV

To assess the presence of mtDNA lesions blocking the progress of polymerases, we performed long PCR experiments to concomitantly amplify a long (8642-bp) and a short (316-bp) mtDNA fragment (Figure 2a). The DNA lesions blocking replication are more likely to be present on a long DNA region than on a short fragment.^25^ The amplification of mtDNA showed that level of the long mtDNA fragment was decreased in the lung tissues of OVA_LPS_-OVA mice compared with the level of saline-sensitized and -challenged (SAL-SAL) mice, whereas the short mtDNA fragment was not changed significantly in all group tested (Figure 2a). The decrease in amplified products of the long mtDNA fragment was markedly restored by treatment with NecroX-5. Consistent with these results, the ratio of long mtDNA fragment/short mtDNA fragment as an index of mtDNA integrity was significantly decreased in lung tissues of OVA_LPS_-OVA mice compared with the levels of SAL-SAL mice. Interestingly, the decreased ratio was substantially restored by the treatment with NecroX-5 (Figure 2b). In addition, mtDNA content was also reduced in lung tissues of OVA_LPS_-OVA mice compared with that of SAL-SAL mice. The decreased level of mtDNA was markedly restored to the similar levels of SAL-SAL mice by treatment with NecroX-5 (Figure 2c). These results suggest that mitochondrial ROS can affect the integrity and content of mtDNA in lung tissues of an asthmatic murine model.

### NecroX-5 reduces protein levels of NLRP3, caspase-1 and IL-1*β* in primary cultured tracheal epithelial cells from OVA_LPS_-OVA mice

**Figure 3:**
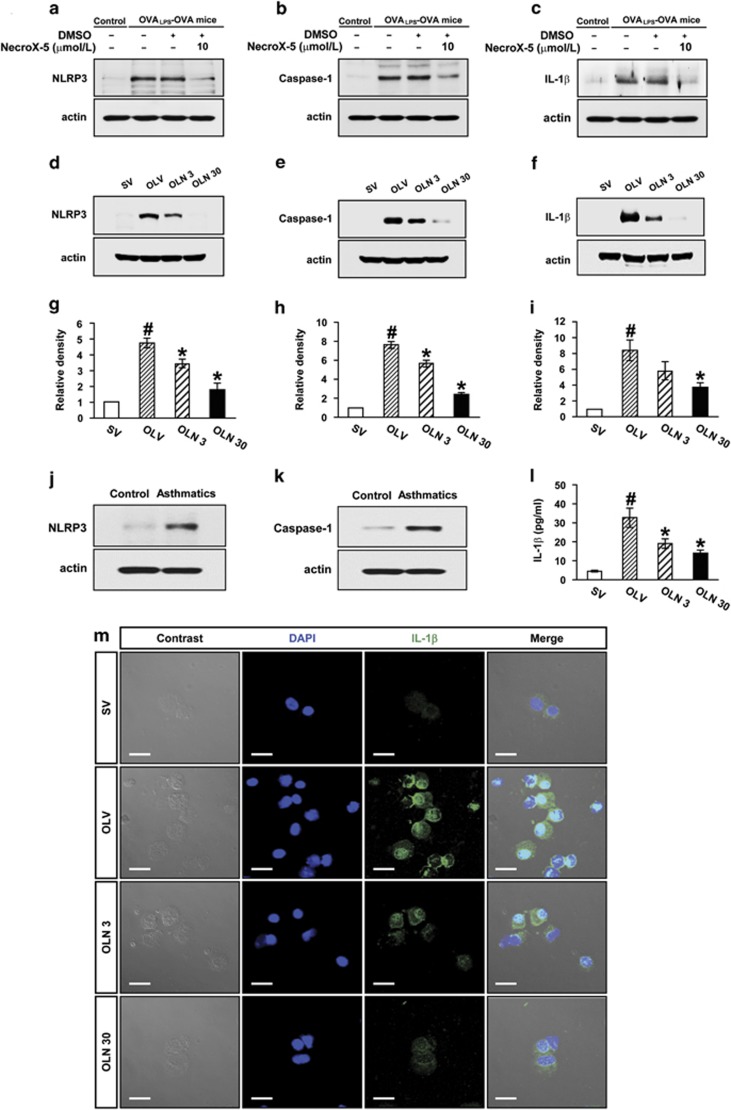
Effects of mitochondrial ROS inhibitor, Necrox-5 on the protein levels of NLRP3, caspase-1 and IL-1*β* in primary cultured tracheal epithelial cells and lung tissues of OVA_LPS_-OVA mice. Representative western blot analysis of NLRP3 (**a**), caspase-1 (**b**) and IL-1*β* (**c**) in primary cultured tracheal epithelial cells of OVA_LPS_-OVA mice. Representative western blots of NLRP3 (**d**), caspase-1 (**e**) and IL-1*β* (**f**) in lung tissues of OVA_LPS_-OVA mice. Densitometric analyses of the bands on films are presented as the relative ratio of NLRP3 (**g**), caspase-1 (**h**) or IL-1*β* (**i**) to actin. The relative ratio of each protein in the lung tissues of SV is arbitrarily presented as 1. Representative western blots of NLRP3 (**j**) and caspase-1 (**k**) in human BAL fluids from asthmatic patients and healthy subjects (*n*=3 per group). (**l**) Enzyme immunoassay of IL-1*β* in BAL fluids of OVA_LPS_-OVA mice. Bars represent mean±S.E.M. from seven mice per group. ^#^*P<*0.05 *versus* SV; *P<0.05 *versus* OLV. (**m**) Representative confocal laser immunofluorescence photomicrography of BAL cells of OVA_LPS_-OVA mice showed the expression of IL-1*β*. The blue fluorescent DAPI stain was used for nuclear localization. The left and the right panels presented phase contrast views and the merger views, respectively. Bars indicate scale of 20 *μ*m

Western blot analyses revealed that levels of NLRP3, caspase-1 and IL-1*β* in primary tracheal epithelial cells from OVA_LPS_-OVA mice were increased significantly compared with the levels in the cells from SAL-SAL mice (Figures 3a–c). Treatment with NecroX-5 significantly reduced the increases of NLRP3, caspase-1 and IL-1*β* levels in primary tracheal epithelial cells from OVA_LPS_-OVA mice.

### Mitochondrial ROS inhibitor reduces activation of NLRP3 inflammasome in lung tissues of OVA_LPS_-OVA mice

Western blot analysis revealed that protein levels of NLRP3, caspase-1 and IL-1*β*, which are the hallmarks of inflammasome activation, in lung tissues of OVA_LPS_-OVA mice were greatly increased at 48 h after the last challenge compared with the levels in SAL-SAL mice (Figures 3d–i). The reduction of mitochondrial ROS by administration of NecroX-5 substantially decreased the expression of these proteins in lung tissues of OVA_LPS_-OVA mice.

In addition, enzyme immunoassay showed that the increased IL-1*β* levels in BAL fluid from OVA_LPS_-OVA mice were significantly decreased by administration of NecroX-5 (Figure 3l). To ascertain the observations, the expression level of IL-1*β* in BAL cells was analyzed by immunofluorescence staining. Immunofluorescence of IL-1*β* was significantly higher in BAL cells from OVA_LPS_-OVA mice than those from SAL-SAL mice. The enhancement of fluorescent intensity in BAL cells of OVA_LPS_-OVA mice was inhibited by administration of NecroX-5 (Figure 3m).

### Activation of NLRP3 inflammasome in asthmatic patients

Western blot analyses showed that the levels of NLRP3 and caspase-1 in BAL fluids from the patients with asthma were significantly higher than the levels in healthy subjects (Figures 3j and k). They were all male persons and their phenotypes were as follows: mean age: 54.0±2.5 (healthy subjects) and 62.0±1.5 (asthmatic patients) (years, mean±S.E.M., *P*=0.066), mean FEV_1_ of asthmatic patients: 76.0±16.2% (of predictive value, mean±S.E.M.).

### Effects of NecroX-5 on cellular changes in BAL fluids from OVA_LPS_-OVA mice

**Figure 4:**
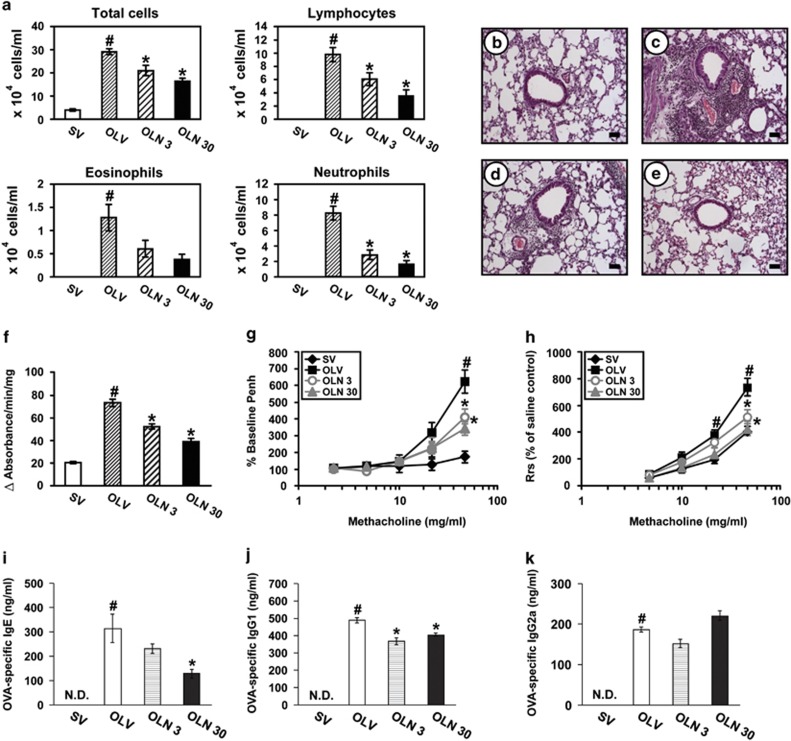
Effects of Necrox-5 on total and differential cell counts in BAL fluids, histological changes, MPO activity, airway hyperresponsiveness and serum levels of OVA-specific immunoglobulins in OVA_LPS_-OVA mice. All parameters were measured at 48 h after the last challenge in SV, OLV, OLN 3 and OLN 30. (**a**) Cellular changes in BAL fluids of OVA_LPS_-OVA mice. Bars represent mean±S.E.M. from seven mice per group. (**b**–**e**) Representative H&E-stained sections of the lungs isolated from SV (**b**), OLV (**c**), OLN 3 (**d**) and OLN 30 (**e**). Bars indicate scale of 20 *μ*m. (**f**) MPO activity in lung tissue homogenates of OVA_LPS_-OVA mice. Airway responsiveness in OVA_LPS_-OVA mice was assessed by both noninvasive (**g**, %Penh) and invasive (**h**, R_rs_) measurements. (**i**–**k**) Changes of the serum levels of OVA-specific IgE (**i**), OVA-specific IgG1 (**j**) and OVA-specific IgG2a (**k**). Bars represent mean±S.E.M. from six or seven mice per group. ND, not detected, ^#^*P*<0.05 *versus* SV; *P<0.05 *versus* OLV

Numbers of total cells, lymphocytes, neutrophils and eosinophils in BAL fluids of OVA_LPS_-OVA mice were increased significantly compared with the numbers of SAL-SAL mice. The increase in numbers of these cells, especially neutrophils, in BAL fluids from OVA_LPS_-OVA mice was significantly reduced by administration of NecroX-5 (Figure 4a).

### NecroX-5 ameliorates pathologic features of OVA_LPS_-OVA mice

Histological assessment showed that numerous inflammatory cells infiltrated into the bronchioles and perivascular regions in the lung of OVA_LPS_-OVA mice (Figure 4c) compared with the SAL-SAL mice (Figure 4b). The OVA_LPS_-OVA mice treated with NecroX-5 showed marked reduction in the infiltration of inflammatory cells in the peribronchiolar and perivascular regions of the lung (Figures 4d and e).

### Inhibition of mitochondrial ROS decreases myeloperoxidase (MPO) activity in lung tissues of OVA_LPS_-OVA mice

MPO is a major constituent of neutrophil cytoplasmic granules, and its activity therefore is a direct measure of the neutrophil presence. As expected, MPO activity was significantly increased in lung tissues of OVA_LPS_-OVA mice (Figure 4f). The increase of MPO activity in lung tissues of OVA_LPS_-OVA mice was substantially reduced by administration of NecroX-5.

### Necrox-5 reduces airway hyperresponsiveness of OVA_LPS_-OVA mice

Airway responsiveness was assessed through both noninvasive and invasive measurements. In OVA_LPS_-OVA mice, the dose-response curve of percent enhanced pause (Penh) or respiratory system resistance (R_rs_) shifted to the left compared with that of SAL-SAL mice (Figures 4g and h). Administration of NecroX-5 reduced substantially the Penh or R_rs_ observed at 50 mg/ml of methacholine in OVA_LPS_-OVA mice.

These results indicate that inhibition of mitochondrial ROS effectively decreases the allergen-induced airway hyperresponsiveness.

### Effects of NecroX-5 on serum level of OVA-specific IgE, IgG1 and IgG2a in OVA_LPS_-OVA mice

Enzyme immunoassays showed the significant increases in OVA-specific IgE, IgG1 and IgG2 levels in the serum of OVA_LPS_-OVA mice compared with the levels in the control mice (Figures 4i–k). The administration of NecroX-5 substantially lowered the serum level of OVA-specific IgE compared with the levels of OVA_LPS_-OVA mice treated with drug vehicle only. In addition, the serum level of OVA-specific IgG1, the Th2-type immunoglobulin was decreased by treatment with NecroX-5, whereas the level of OVA-specific IgG2a was not affected by NecroX-5, although the level of IgG2a showed the increased tendency on the treatment with 30 mg/kg of NecroX-5 in OVA_LPS_-OVA mice.

### NecroX-5 lowers the production of inflammatory cytokines in OVA_LPS_-OVA mice

**Figure 5:**
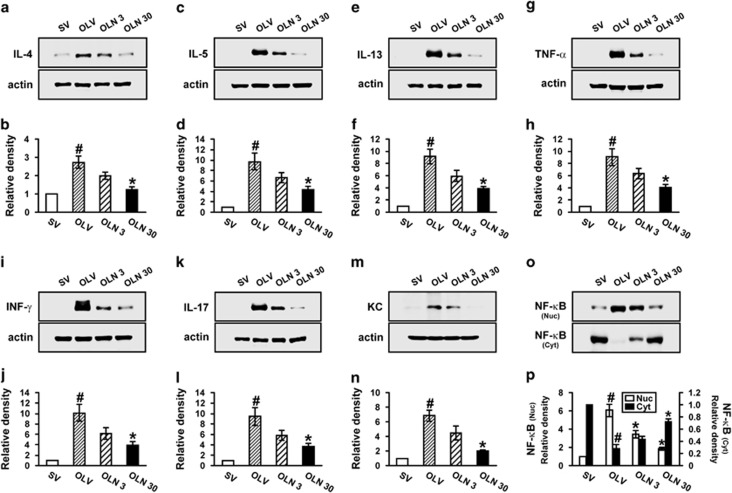
Effects of NecroX-5 on the protein levels of various inflammatory mediators and the activation of NF-*κ*B/I*κ*B*α* in lung tissues of OVA_LPS_-OVA mice. Sampling was performed at 48 h after the last challenge in SV, OLV, OLN 3 and OLN 30. Representative western blots of IL-4 (**a**), IL-5 (**c**), IL-13 (**e**), TNF-*α* (**g**), IFN-*γ* (**i**), IL-17 (**k**) and KC (**m**) in lung tissues. (**b**, **d**, **f**, **h**, **j**, **l**, **n**) Densitometric analysis of the bands on films is presented as the relative ratio of each protein to actin. (**o**) Representative western blot of NF-*κ*B p65 in lung tissues. (**p**) Densitometric analyses of the bands on films are presented as the relative ratio of NF-*κ*B p65 level in OLV, OLN 3 or OLN 30 to the level in SV. The relative ratio of each protein in the lung tissues of SV is arbitrarily presented as 1. Bars represent mean±S.E.M. from seven mice per group. ^#^*P*<0.05 *versus* SV; *P<0.05 *versus* OLV

To investigate whether mitochondrial ROS evokes the inflammatory responses in allergic airway inflammation, we examined the effects of NecroX-5 on the levels of inflammatory cytokines in the lung of OVA_LPS_-OVA mice. Western blot analyses showed that levels of IL-4, IL-5, IL-13, TNF-*α*, IFN-*γ*, IL-17 and KC protein in lung tissues of OVA_LPS_-OVA mice were significantly increased compared with the levels of SAL-SAL mice (Figures 5a–n). Administration of NecroX-5 reduced significantly the OVA-induced increases of IL-4, IL-5, IL-13, TNF-*α*, IFN-*γ*, IL-17 and KC protein in lung tissues.

### NecroX-5 prevents nuclear translocation of NF-*κ*B p65 in lung tissues of OVA_LPS_-OVA mice

In western blotting data, the level of nuclear nuclear factor-*κ*B (NF-*κ*B) p65 in lung tissues of OVA_LPS_-OVA mice was significantly increased compared with the level in SAL-SAL mice (Figures 5o and p). The increase of nuclear NF-κB p65 was significantly reduced by administration of NecroX-5.

### NecroX-5 reduces allergic airway inflammation, bronchial hyperresponsiveness, activation of NLRP3 inflammasome and generation of mitochondrial ROS in the lung of house dust mite (HDM)-instilled mice

**Figure 6:**
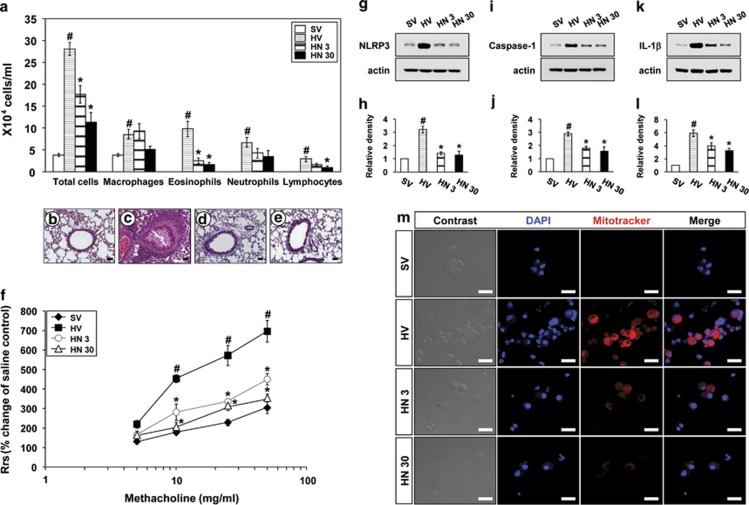
Effects of NecroX-5 on total and differential cell counts in BAL fluids, histological changes, airway hyperresponsiveness, protein levels of NLRP3, IL-1*β* and caspase-1, and generation of mitochondrial ROS in the lung of HDM-instilled mice. All parameters were measured at 48 h after the last challenge in saline-instilled mice administered drug vehicle (SV), HDM-instilled mice administered drug vehicle (HV), HDM-instilled mice administered 3 mg/kg of NecroX-5 (HN 3) and HDM-instilled mice administered 30 mg/kg of NecroX-5 (HN 30). (**a**) Cellular changes in BAL fluids of HDM-instilled mice. Bars represent mean±S.E.M. from six mice per group. (**b**–**e**) Representative H&E-stained sections of the lungs isolated from SV (**b**), HV (**c**), HN 3 (**d**) and HN 30 (**e**). Bars indicate scale of 20 *μ*m. (**f**) Airway responsiveness in HDM-instilled mice was assessed by invasive measurement (R_rs_). (**g**–**l**) Representative western blots for NLRP3 (**g**), caspase-1 (**i**) and IL-1*β* (**k**) in lung tissues and densitometric analysis. Bars represent mean±S.E.M. from six mice per group. (**m**) Representative confocal laser immunofluorescence photomicrography of BAL cells showed the localization of mitochondrial ROS (red fluorescence views) in the cells. The blue fluorescent DAPI stain was used for nuclear localization. The left and the right panels presented phase contrast views and the merger views, respectively. Bars indicate scale of 20 *μ*m. ^#^*P*<0.05 *versus* SV; *P<0.05 *versus* HV

Numbers of total cells, eosinophils, neutrophils and lymphocytes in BAL fluids of HDM-instilled mice were increased significantly compared with the numbers of saline-instilled mice administered drug vehicle (SV). The increase in the numbers of these cells, especially eosinophils, in BAL fluids from HDM-instilled mice was significantly reduced by administration of NecroX-5 (Figure 6a). Histological assessment showed that numerous inflammatory cells infiltrated into the bronchioles and perivascular regions in the lung of HDM-instilled mice (Figure 6c) compared with the SV mice (Figure 6b). The HDM-instilled mice treated with NecroX-5 showed marked reduction in the infiltration of inflammatory cells in the peribronchiolar and perivascular regions of the lung (Figures 6d and e). Airway responsiveness was assessed by R_rs_ measurement. In HDM-instilled mice, the methacholine dose-response curve of R_rs_ shifted to the left compared with that in SV mice (Figure 6f). Administration of NecroX-5 reduced substantially the R_rs_ observed at 10–50 mg/ml of methacholine in HDM-instilled mice. Western blot analysis revealed that protein levels of NLRP3, caspase-1 and IL-1*β* in lung tissues of HDM-instilled mice were greatly increased at 48 h after the last challenge compared with the levels in SV mice (Figures 6g–l). The administration of NecroX-5 substantially decreased the expression of these proteins in lung tissues of HDM-instilled mice. Intensity of fluorescence was significantly higher in BAL cells (Figure 6m) from HDM-stilled mice than those from SV mice. The enhancement of fluorescence intensity in BAL cells from HDM-instilled mice was inhibited by treatment with NecroX-5.

### Effects of blockade of IL-1*β* on cellular changes in BAL fluids, airway hyperresponsiveness and levels of inflammatory cytokines in the lung of OVA_LPS_-OVA mice and OVA_LPS_-OVA IL-1R knock-out (KO) mice

**Figure 7:**
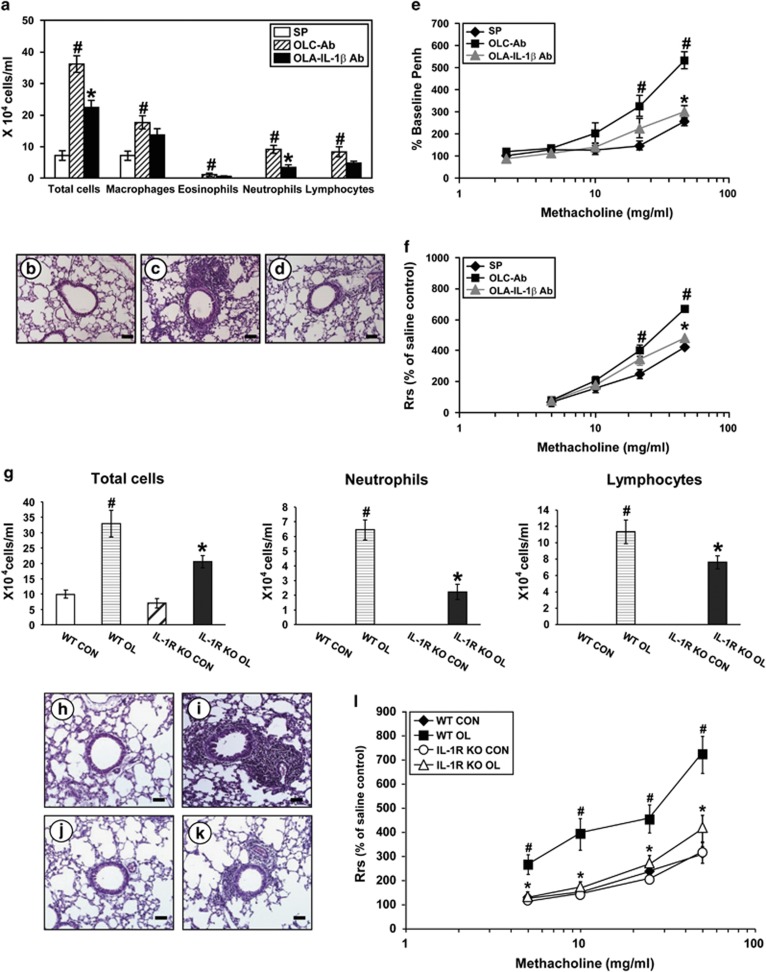
Effects of blockade of IL-1*β* on total and differential cell counts in BAL fluids, histological changes and airway hyperresponsiveness in the lung of OVA_LPS_-OVA mice and OVA_LPS_-OVA IL-1R KO mice. All parameters were measured at 48 h after the last challenge in SAL-SAL mice administered with PBS (SP), OVA_LPS_-OVA mice administered with isotype control antibody (OLC-Ab), OVA_LPS_-OVA mice administered with an anti-IL-1*β*-neutralizing antibody (OLA-IL-1*β* Ab), SAL-SAL WT mice (WT CON), OVA_LPS_-OVA WT mice (WT OL), SAL-SAL IL-1R KO mice (IL-1R KO CON) and OVA_LPS_-OVA IL-1R KO mice (IL-1R KO OL). (**a**) Cellular changes in BAL fluids of OVA_LPS_-OVA mice. Bars represent mean±S.E.M. from seven mice per group. (**b**-**d**) Representative H&E-stained sections of the lungs isolated from SP (**b**), OLC-Ab (**c**) and OLA-IL-1*β* Ab (**d**). Bars indicate scale of 20 *μ*m. (**e** and **f**) Airway responsiveness in OVA_LPS_-OVA mice was assessed by both noninvasive (**e**, %Penh) and invasive (**f**, R_rs_) measurements. Bars represent mean±S.E.M. from seven mice per group. (**g**) Cellular changes in BAL fluids of IL-1R KO or WT mice. Bars represent mean±S.E.M. from five mice per group. (**h**–**k**) Representative H&E-stained sections of the lungs isolated from WT CON (**h**), WT OL (**i**), IL-1R KO CON (**j**) and IL-1R KO OL (**k**). Bars indicate scale of 50 *μ*m. (**l**) Airway responsiveness in IL-1R KO or WT mice was assessed by invasive measurement (R_rs_). Bars represent mean±S.E.M. from five mice per group. ^#^*P*<0.05 *versus* SP or WT CON; *P<0.05 *versus* OLC-Ab or WT OL

In order to verify the action mechanism of NecroX-5, that is, whether or not the anti-asthmatic effect of NecroX-5 works via suppression of inflammasome activation, we evaluated the role of IL-1*β* on changes in BAL cells, histologic features, airway hyperresponsiveness and protein levels of pro-inflammatory cytokines using an anti-IL-1*β-*neutralizing antibody or IL-1R KO mice. The increased numbers of total cells, lymphocytes, neutrophils and eosinophils in OVA_LPS_-OVA mice were substantially reduced by neutralization of IL-1*β* with the antibody (Figure 7a). In addition, the OVA_LPS_-OVA mice treated with the anti-IL-1*β-*neutralizing antibody showed a marked reduction in infiltration of numerous inflammatory cells into peribronchiolar and perivascular regions on histologic examination (Figures 7b–d).

Airway responsiveness assessed by both noninvasive and invasive measurements, a percent increase of Penh or R_rs_ in response to increasing doses of methacholine, was revealed as follows: in OVA_LPS_-OVA mice, the percent Penh or R_rs_ produced by methacholine was significantly increased compared with the levels of SAL-SAL mice (Figures 7e and f). Treatment with the anti-IL-1*β-*neutralizing antibody significantly reduced the Penh and the R_rs_ at 50 mg/ml of methacholine compared with that of OVA_LPS_-OVA mice.

**Figure 8:**
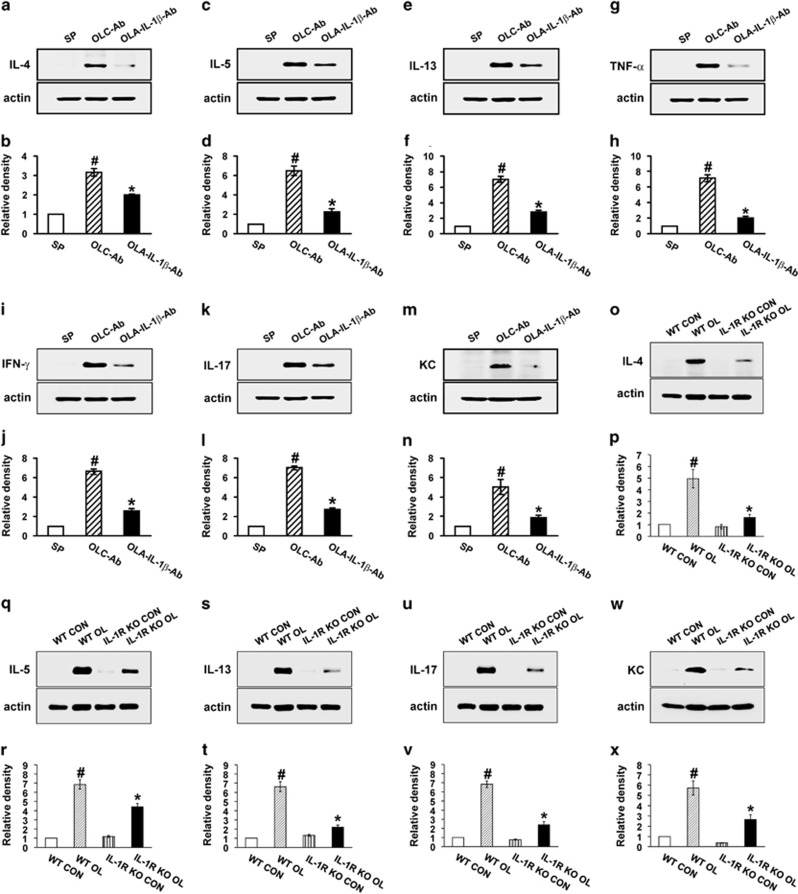
Effects of blockade of IL-1*β* on the protein levels of various inflammatory mediators in lung tissues of OVA_LPS_-OVA mice and OVA_LPS_-OVA IL-1R KO mice. Sampling was performed at 48 h after the last challenge in SP, OLC-Ab, OLA-IL-1*β* Ab, WT CON, WT OL, IL-1R KO CON,and IL-1R KO OL. Representative western blots of IL-4 (**a** and **o**), IL-5 (**c** and **q**), IL-13 (**e** and **s**), TNF-*α* (**g**), IFN-*γ* (**i**), IL-17 (**k** and **u**) and KC (**m** and **w**) in lung tissues. (**b**, **d**, **f**, **h**, **j**, **l**, **n**, **p**, **r**, **t**, **v**, **x**) Densitometric analysis of the bands on films is presented as the relative ratio of each protein to actin. The relative ratio of each protein in the lung tissues of SP or WT CON is arbitrarily presented as 1. Bars represent mean±S.E.M. from five or seven mice per group. ^#^*P*<0.05 *versus* SP or WT CON; *P<0.05 *versus* OLC-Ab or WT OL

The protein levels of IL-4, IL-5, IL-13, TNF-*α*, IFN-*γ*, IL-17 and KC protein in lung tissues of OVA_LPS_-OVA mice were markedly increased compared with the levels in SAL-SAL mice (Figures 8a–n). Administration of the anti-IL-1*β-*neutralizing antibody significantly reduced the increases of IL-4, IL-5, IL-13, TNF-*α*, IFN-*γ*, IL-17 and KC protein in lung tissues.

Consistent with these findings, OVA_LPS_-OVA IL-1R KO mice showed the significantly reduced asthmatic manifestations including the number of airway inflammatory cells, pathologic changes and airway hyperresponsiveness compared with the levels of OVA_LPS_-OVA wild-type (WT) mice (Figures 7g–l). In addition, the IL-1R KO mice showed the lower levels of pro-inflammatory mediators such as IL-4, IL-5, IL-13, IL-17 and KC in lung tissues than the levels of OVA_LPS_-OVA WT mice after OVA challenges (Figures 8o–x).

These results indicate that blockade of IL-1*β* action attenuates inflammatory responses and airway hyperresponsiveness in OVA_LPS_-OVA mice.

## Discussion

In this study, we demonstrate for the first time the role of mitochondrial ROS in the development of allergic inflammation using an OVA and LPS-induced mouse model of neutrophil-dominant allergic airway disease and HDM-induced allergic asthmatic murine model. Moreover, the molecular bases of action of a novel mitochondrial ROS inhibitor, NecroX-5, were evaluated in this pathologic situation, in which NecroX-5 attenuated the asthmatic features including airway inflammation and hyperresponsiveness through the modulation of NLPR3 inflammasome activation that leads to the production of IL-1*β*.

It is well known that one of the causative mechanisms of airway inflammation and airway obstruction is oxidative stress or tilt in the delicate balance of the cellular redox state.^1^ Moreover, allergen-activated and recruited inflammatory cells such as eosinophils, macrophages, monocytes and neutrophils from asthmatic patients produce more ROS than those from normal subjects do.^26^ The constitutive airway cells such as epithelial cells are also a potential source of ROS.^27^ In addition to cellular sources of ROS, several asthma mediators including lipid mediators, chemokines, adhesion molecules and eosinophil granule proteins are potential stimuli of ROS production.^28, 29, 30^ Thus, antioxidant treatment of allergic asthmatic inflammation has long been a subject of therapeutic strategy. Despite plentiful data from animal studies using antioxidants,^31,32^ previous human studies have yielded disappointing results with the effects of antioxidant supplementation in allergic airway inflammation, such as vitamin C, vitamin E and flavonoids. However, as these compounds do not significantly accumulate within mitochondria, their effectiveness remains limited.^33^ Although endogenous sources of intracellular ROS include the NADPH oxidases (NOXs), the Ero1–DPI oxidative folding system in the endoplasmic reticulum, and the mitochondrial electron transport chain,^34^ there is no available evidence to date that shows for which source has a major role in the pathogenesis of bronchial asthma. In this study, our results showed that OVA challenge increases cellular ROS generation in BAL cells. More intriguingly, we also found that mitochondrial ROS generation is significantly increased in BAL cells and primary cultured tracheal epithelial cells from OVA_LPS_-OVA mice or HDM-instilled mice and that a ROS inhibitor, NecroX-5, markedly reduces total and mitochondrial ROS generation in the mice. Moreover, our data revealed that enhanced airway hyperresponsiveness and inflammation in OVA_LPS_-OVA mice or HDM-instilled mice were substantially suppressed by administration of Necrox-5. These findings indicate that bronchial asthma can be aggravated, at least in part, by the induction of mitochondrial ROS generation in airway inflammatory cells and epithelial cells and that a novel antioxidant, NecroX-5, works well targeting mitochondrial ROS in airway inflammation.

Inflammasomes are a group of protein complexes that recognize a diverse set of inflammation-inducing stimuli, pathogen-associated molecular pattern molecules (PAMPs) and damage-associated molecular pattern molecules (DAMPs) and that control production of important pro-inflammatory cytokines such as IL-1*β* and IL-18.^35^ Whereas the absent in melanoma 2 and NLRC4 inflammasomes are activated only by specific PAMPs, double-stranded DNA and specific bacterial proteins, respectively,^36,37^ NLRP3 is activated by a large variety of signals, including PAMPs, DAMPs and bacterial toxins.^15, 16, 17^ Two common events that are required for these activators of the NLRP3 inflammasome are potassium efflux and ROS generation. Scavenging of ROS blocks NLRP3 inflammasome activation in response to a wide variety of stimuli.^15,16,38^ It was initially postulated that the source of ROS is from the phagosome-associated NADPH oxidase.^15^ However, a number of subsequent studies have demonstrated that the NADPH oxidase is dispensable for NLRP3 inflammasome activation,^39,40^ suggesting that the source of the ROS may be of mitochondrial origin. In addition, treatment of macrophages with Mito-TEMPO, a scavenger of mitochondrial ROS, resulted in inhibition of NLRP3 inflammasome activation.^13^ Furthermore, mtDNA is very sensitive to oxidative stress up to 50-fold more compared with nuclear DNA.^41^ Severe damaged and not properly repaired mtDNA are known to be released to cytoplasm from mitochondria, which function as a coactivator in the NLRP3-dependent activation of caspase-1.^42^ These damaged mtDNA fragments accumulated in cytoplasm have been called as mitochondrial DAMPs. In our OVA_LPS_-OVA mice or HDM-instilled mice, the protein expression levels of NLRP3 and caspase-1 were significantly increased in lung tissues and the primary cultured tracheal epithelial cells. In addition, active form of IL-1*β* was highly expressed in lung tissues and primary cultured tracheal epithelial cells, as well as various BAL cells from OVA_LPS_-OVA mice. Furthermore, we revealed that the protein expression levels of NLRP3 and caspase-1 were also increased in BAL fluids from asthmatic patients compared with the levels of healthy subjects. Treatment with NecroX-5 substantially reduced the increases in protein levels of NLRP3, caspase-1 and IL-1*β* in the lung tissues of OVA_LPS_-OVA mice and HDM-instilled mice. In addition, our data showed that mtDNA was significantly damaged and reduced in lung tissues of OVA_LPS_-OVA mice and that the integrity and content of mtDNA were restored by the treatment with NecroX-5. These results suggest that NLRP3 inflammasome assembly is activated under airway inflammatory conditions, which consequently induces IL-1*β* production and release. They also suggest that mitochondrial ROS generation and/or the mtDNA damage is closely associated with NLRP3 inflammasome activation in bronchial asthma. In addition, airway epithelial cells can be one of source cells in which the inflammasome activation occurs.

IL-1 family cytokines, such as IL-1*α* and IL-1*β*, are involved in multiple aspects of immune and inflammatory responses.^43^ There is increasing evidence that IL-1 contributes to allergic diseases by promoting mast cell activation and Th2 cytokine production.^44^ Indeed, elevated levels of IL-1*β* have been found in the BAL fluid from patients with asthma.^45^ Classically, IL-1*β* is synthesized as a precursor of IL-1*β* (pro-IL-1*β*) by blood monocytes, tissue macrophages and dendritic cells after activation by pro-inflammatory stimuli such as IL-1, TNF or TLR ligands^43^ and needs to be processed to be active. Pro-IL-1*β* is cleaved by the protease caspase-1, which is activated through an inflammasome.^17^ Importantly, recent studies have revealed that NLRP3 inflammasome activation, leading to IL-1 production, is critical in allergic lung inflammation and that exposure of Nlrp3−/− and WT mice to urban particulate matter demonstrates NLRP3-dependent production of IL-1*β* in the lung and airway neutrophilia.^18,46^ Our current data from OVA_LPS_-OVA-induced asthmatic mice *in vivo* and primary cultured epithelial cells *in vitro* showed that both cleaved caspase-1 and mature IL-1*β* levels were increased, indicating that NLRP3 inflammasome is functional. We also observed the presence of IL-1*β* in various BAL cells, *in situ* positive IL-1*β* staining from BAL fluid specimens. These results support our contention that NLRP3 inflammasome is active in response to inflammatory stimuli, subsequently producing mature IL-1*β* in airway inflammatory cells and epithelial cells in a classic manner. In addition, for the confirmation whether the mature IL-1*β* contributes to the development of asthmatic symptoms, that is, airway inflammation and hyperresponsiveness, we evaluated the effects of blockade of IL-1*β* on the asthmatic features. Consequently, blockade of IL-1*β* by treatment with an anti-IL-1*β-*neutralizing antibody or the use of IL-1R KO mice successfully inhibited the increased airway inflammation and hyperresponsivenss in our current animal model.

NF-*κ*B, a multiprotein complex, is involved in the early cellular defense reactions in higher organisms and has a pivotal role in immune and inflammatory responses.^47^ Development of oxidant/antioxidant imbalance in asthma leads to the activation of this redox-sensitive transcription factor, NF-*κ*B.^48^ ROS have also been directly implicated as second messengers in the activation of NF-*κ*B, based on the ability to activate NF-*κ*B by oxidation of a cysteine-SH group or by ubiquitination and proteolysis of I*κ*B.^49^ Consistent with these observations, our results showed that NF-*κ*B levels in nuclear protein extracts from lung tissues are substantially increased in the OVA_LPS_-OVA-induced neutrophilic allergic airway disease. In addition, the increase of various pro-inflammatory mediators including Th2 cytokines, IL-17, KC, TNF-*α* and IL-1*β* was occurred in our OVA_LPS_-OVA-induced murine model of allergic airway disease. Administration of NecroX-5 resulted in a significant reduction of NF-*κ*B translocation into the nucleus, accompanying reduction in the expression of Th2 cytokines, IL-17, KC, TNF-*α* and IL-1*β* in the lung of OVA_LPS_-OVA mice, which are linked to asthmatic symptoms. These results indicate that inhibition of mitochondrial ROS reduces the traits of asthmatic symptoms through modulation of NF-*κ*B activation as well as control of NLRP3 inflammasome.

Conclusively, this study using both murine models of allergic asthma, that is, OVA_LPS_-OVA mice and HDM-instilled mice suggests that mitochondrial ROS have a critical role in the pathogenesis of allergenic airway inflammation through the modulation of NLRP3 inflammasome activation, especially in bronchial epithelial cells as an immune responder, providing a novel concept of therapeutic strategy for allergen-induced airway disorders.

## Materials and Methods

### Animals and experimental protocol

Female C57BL/6 mice, 8 to 10 weeks of age and free of murine-specific pathogens, were obtained from the Orient Bio Inc. (Seoungnam, Korea). In addition, female C57BL/6 IL-1R KO mice and WT mice, 8 to 10 weeks of age and free of murine-specific pathogens, were obtained from the Jackson Laboratory (Sacramento, CA, USA). They were housed throughout the experiments in a laminar flow cabinet, and were maintained on standard laboratory chow *ad libitum*. All experimental animals used in this study were under a protocol approved by the Institutional Animal Care and Use Committee of the Chonbuk National University. For the neutrophilic asthma model, mice were sensitized intranasally with OVA and LPS (OVA_LPS_; 10 *μ*g of OVA combined with 1 *μ*g of LPS in saline; Sigma-Aldrich, St. Louis, MO, USA) on days 1, 2, 3 and 14. On days 21, 22 and 23 after the initial sensitization, the mice were challenged for 30 min with an aerosol of 3% (wt/vol) OVA in saline (or with saline as a control) using an ultrasonic nebulizer (NE-U17, Omron, Kyoto, Japan). In case of HDM-induced asthma models, female C57BL/6 mice (Orient Bio Inc.) were sensitized intratracheally with HDM extract (100 *μ*g, Demarophagoides pteronyssius, GREER Laboratories, Lenoir, NC, USA) on days 1 and 7. On day 14 after the initial sensitization, the mice were challenged intratracheally with 100 *μ*g of HDM extract in saline (or with saline as a control). BAL was performed at 48 h after the last challenge with OVA or HDM in mice. At the time of lavage, the mice were killed by cervical dislocation. The chest cavity was exposed to allow for expansion, after which the trachea was carefully intubated and the catheter secured with ligatures. Prewarmed 0.9% NaCl solution was slowly instilled into the lung and withdrawn. The collected solutions were pooled and then kept at 4 °C. A part of each pool was then centrifuged, and the supernatants were kept at −70 °C until use. Total cell numbers were counted with a hemocytometer. Smears of BAL cells were prepared by cytospin (Thermo Electron, Waltham, MA, USA) and stained with Diff-Quik solution (Dade Diagnostics of Puerto Rico Inc., Aguada, Puerto Rico) in order to examine cell differentials. Two independent, blinded investigators counted the cells using a microscope. Approximately 400 cells were counted in each of four different random locations. Inter-investigators variation was <5%. The mean number from the two investigators was used to estimate the cell differentials.

### Administration of a mitochondrial ROS inhibitor, NecroX-5 and an anti-IL-1*β-*neutralizing antibody

**Figure 9:**
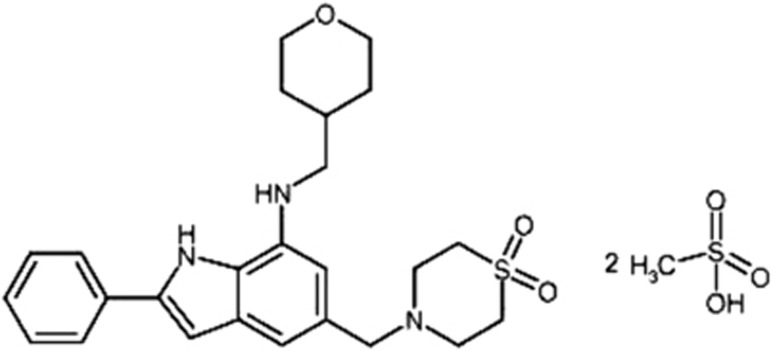
Structure of NecroX-5

NecroX-5 (3 or 30 mg/kg body wt/day, Enzo Life Sciences, Farmingdale, NY, USA; Figure 9) diluted with distilled water was administered by intraperitoneal injection two times to each treated animal, once at 1 h before the first challenge with OVA and the second time at 6 h after the last challenge in OVA_LPS_-OVA mice. Anti-IL-1*β-*neutralizing antibody or isotype control monoclonal antibody (100 *μ*g/kg body wt per day, eBioscience, San Diego, CA, USA) was administered intravenously two times to each animal, once at 1 h before the first challenge with OVA and the second time at 6 h after the last challenge in OVA_LPS_-OVA mice.

### Measurement of total cellular ROS and mitochondrial ROS

Total cellular ROS were measured as described previously with a modification.^50^ To measure intracellular ROS level, cells were incubated for 10 min at room temperature with PBS containing 3.3 *μ*mol/l *2′,7′*-dichlorofluorescein-diacetate (DCF-DA) (Invitrogen, Carlsbad, CA, USA). As for mitochondrial ROS, cells were stained with MitoTracker Red CM-H_2_ROS (Invitrogen). Each labeled ROS in cells was then immediately observed under FACSCalibur apparatus (BD Biosciences, San Jose, CA, USA). The data were analyzed with a CellQuest Pro software (BD Biosciences).

### Confocal microscopy

BAL cells or primary cultured tracheal epithelial cells were attached to coverglass-bottom dish for 1 h and then stained with Mitotracker Red CM-H_2_ROS (Invitrogen) in the dark at room temperature. After 30 min, cells were washed with PBS and analyzed using a confocal laser scanning microscope (LSM 510 META, Carl Zeiss, Jena, Germany).

### Measurement of integrity and content of mtDNA

First, to perform long PCR experiments, total DNA was extracted from lung tissue samples using DNAZOL reagent according to manufacturer's protocol (Gibco/Life Technologies, Carlsbad, CA, USA). This long PCR technique is based on the amplification of a long (8642-bp) and a short (316-bp) mtDNA fragment. The primers used were as follows: short mtDNA fragment, sense: 5′-CGACAGCTAAGACCCAAACTGGG-3′, antisense: 5′-CCCATTT CTTCCCATTTCATTGGC-3′, and long mtDNA fragment, sense: 5′-TACTAGTCCGCGAGCCTTCAAAGC-3′, antisense: 5′-GGGTGATCTTTGTTTGCGGGT-3′. PCR reactions were performed in a thermocycler (GeneAmps PCR System 2400, Applied Biosystems, Foster City, CA, USA). The thermocycler profile for short mtDNA fragment included initial denaturation at 94 °C for 2 min, denaturation at 95 °C for 45 s, annealing at 61 °C for 10 s and extension at 68 °C for 8 min, and final extension at 68 °C for 7 min, whereas for long mtDNA fragment, the profile is follows: initial denaturation at 75 °C for 2 min and 94 °C and 1 min, denaturation at 94 °C for 15 s, annealing at 59 °C for 30 s and extension at 65 °C for 12 min, and final extension at 72 °C for 10 min. The amplified PCR products were electrophoresed using 1.6% agarose gels stained with ethidium bromide. DNA bands were visualized using Fuji film LAS-3000 (Fuji film, Tokyo, Japan) under ultraviolet transillumination.

For the measurement of the content of mtDNA, mitochondria were isolated by differential centrifugation in lung tissues of mice. mtDNA was isolated using Mit minochondrial DNA isolation kit (Biovision Inc., Mountain view, CA, USA) and dissolved in a Tris-EDTA (10 mmol/l Tris·HCl (pH 8.0) and 1 mmol/l EDTA) buffer. Samples were quantified to a final concentration of 1 ng/*μ*l. Protein levels were determined using Bradford reagent (Bio-Rad Laboratories, Hercules, CA, USA).

### BAL procedure

Three asthmatic patients and three healthy subjects were pretreated intramuscularly with atropine (0.5 mg), and 4% lidocaine was used as a local anesthetic immediately before the BAL procedure, which was performed in the segmental bronchus of the right middle lobe. Fifty milliliters of warm 0.9% NaCl solution was instilled into the bronchial tree five times, followed by gentle suction with a negative pressure 80–100 mm Hg using a flexible fiberoptic bronchoscope (Olympus BF-260; Olympus Optical Co. Ltd, Tokyo, Japan). No complications occurred during the BAL procedures. The total volume of recovered fluids was measured. The aspirated fluid was collected into sterile siliconized containers at 4 °C and then filtered through sterile gauze and separated into its fluid and cellular components by centrifugation at 400 × *g* for 10 min at 4 °C. The study was approved by Institutional Review Board of Choubuk National University Hospital (IRB file No. 2009-02-01).

### Western blot analysis

Lung tissues or BAL cells were homogenized in the presence of protease inhibitor cocktail (Sigma-Aldrich), and protein concentrations were determined using Bradford reagent (Bio-Rad Laboratories). Samples were loaded onto a SDS-PAGE gel. After electrophoresis at 120 V for 90 min, proteins were transferred to polyvinylidene difluoride membranes (Bio-Rad Laboratories) at 250 mA for 90 min by a wet transfer method. Nonspecific sites were blocked with 5% non-fat dry milk in Tris-buffered saline Tween 20 (25 mmol/l Tris pH 7.5, 150 mmol/l NaCl, 0.1% Tween 20) for 1 h, and the blots were then incubated overnight at 4 °C with an anti-NLRP3 antibody (Adipogen, San Diego, CA, USA), anti-caspase-1 antibody (Santa Cruz Biotechnology, Santa Cruz, CA, USA), anti-IL-4 antibody (Serotec Ltd, Oxford, UK), anti-IL-5 antibody (Santa Cruz Biotechnology), anti-IL-13 antibody (R&D Systems, Minneapolis, MN, USA), anti-IL-17 antibody (R&D Systems), anti-KC antibody (BioVision, Mountain View, CA, USA), anti-IFN-*γ* antibody (Santa Cruz Biotechnology), anti-IL-1*β* antibody (Thermo Scientific, Waltham, MA, USA), anti-TNF-*α* antibody (Thermo Scientific) and anti-actin antibody (Sigma-Aldrich). Anti-rabbit or anti-mouse horseradish peroxidase-conjugated-IgG (Cell Signaling Technology, Beverly, MA, USA) was used to detect binding of antibodies. The binding of the specific antibody was visualized using Fuji film LAS-3000 (Fuji film) after treating with enhanced chemiluminescence system reagents (GE Healthcare, Little Chalfont, Buckinghamshire, UK). Densitometric analysis was performed on the relative intensity of each band using the Multi Gauge program, version 3.0 (Fuji film). For the quantification of specific bands, the square with the same size was drawn around each band to measure the density and then the value was adjusted by the density of the background near that band. The results of densitometric analysis were expressed as a relative ratio of the target protein to reference protein. The relative ratio of the target protein of control group is arbitrarily presented as 1.

### Histology and immunohistochemistry

Forty-eight hours after the last OVA challenge, mice were killed. The lung and trachea of mice were filled with 10% (v/v) neutral buffered formalin intratracheally and then were removed from the mice. For fixation, the neutral buffered formalin was also used. Specimens were dehydrated and embedded in paraffin. For histological examination, 4-*μ*m sections of fixed embedded tissues were cut on a Leica model 2165 rotary microtome (Leica Microsystems Nussloch GmbH, Wetzlar, Germany), placed on glass slides, deparaffinized and stained sequentially with hematoxylin 2 and eosin-Y (Richard-Allan Scientific, Kalamazoo, MI, USA). Stained slides were analyzed using a light microscope (Axio Imager M1, Carl Zeiss) under identical conditions, including magnification ( × 20), gain, camera position, and background illumination.^51^

### Measurement of IL-1*β* in BAL fluids

Levels of IL-1*β* in BAL fluids were quantified in supernatants of BAL fluids by enzyme immunoassay according to the manufacturer's protocol (R&D Systems). Sensitivity for IL-1*β* assays was 3.0 pg/ml.

### Measurement of OVA-specific IgE, IgG1 and IgG2a in serum

Levels of OVA-specific IgE, IgG1 and IgG2a in serum were quantified by enzyme immunoassay according to the manufacturer's protocol (eBioscience). Sensitivities for IgE, IgG1 and IgG2a assays were 4.0, 3.13 and 3.90 ng/ml, respectively.

### Immunofluorescence staining for IL-1*β*

Smeared BAL cells were fixed with ice cold methanol, permeabilized in PBS containing 0.25% Triton X-100 for 10 min at room temperature and washed three times with PBS. Subsequently, after antigen retrieval for 15 min at 37 °C in proteinase K (Dako, Glostrup, Denmark), nonspecific bindings were blocked with 1% bovine serum albumin (BSA; Sigma-Aldrich) in PBS containing 0.05% Tween 20 (0.05% PBS-T) for 1 h. Specimens were then incubated in a humidified chamber for 2 h at room temperature with an anti-IL-1*β* antibody (Santa Cruz Biotechnology). For the detection of binding antibody to IL-1*β*, Alexa Fluor 488 (green) labeled donkey anti-goat IgG (Invitrogen) in 1% BSA were loaded for 1 h at room temperature in the dark. After the specimens were washed, nuclei were stained using 4'-6-diamidino-2-phenylindole (DAPI; Invitrogen). Stained cells were mounted on slides using fluorescent mounting medium (Golden Bridge International, Inc., Mukilteo, WA, USA) and visualized using a confocal microscope (Zeiss LSM 510 Meta, Carl Zeiss) equipped with a C-Apochromat 63 × /1.20 W Korr UV-VIS-IR M27 water immersion objective. Phase contrast microscopy of each group was used for morphological analysis of BAL cells.

### MPO assay

MPO was extracted from each homogenized lung tissue sample by suspending the sample in 0.5% hexadecyltrimethylammonium bromide (Sigma Chemical Co., St. Louis, MO, USA) in 50 mmol/l potassium phosphate buffer, pH 6.0, before sonication in an ice bath for 20 s. The samples were freeze-thawed two times, after which sonication was repeated. Suspensions were then centrifuged at 13 200 r.p.m. for 15 min, and the supernatant was assayed. MPO activity was determined by mixing 1 *μ*l of supernatant with 299 *μ*l of the above potassium phosphate buffer containing 0.167 mg/ml o-dianisidine dihydrochloride (Sigma Chemical Co.) and 0.0005% hydrogen peroxide (Sigma Chemical Co.). The change in absorbance at 450 nm was measured. MPO activity was then derived from the observed change in absorbance per minute. The activity was expressed as the observed change in absorbance per minute per milligram of protein.

### Isolation and primary culture of murine tracheal epithelial cells

Murine tracheal epithelial cells were isolated under sterile conditions as described previously.^52^ The epithelial cells were seeded onto 60-mm collagen-coated dishes for submerged culture. The growth medium, DMEM (Invitrogen), containing 10% fetal bovine serum, penicillin, streptomycin and amphotericin B was supplemented with insulin, transferrin, hydrocortisone, phosphoethanolamine, cholera toxin, ethanolamine, bovine pituitary extract and BSA. The cells were maintained in a humidified 5% CO_2_ incubator at 37 °C until they adhered.

### Necrox-5 treatment on murine tracheal epithelial cells from OVA_LPS_-OVA mice

Cells were seeded in culture dishes and grown until 70% confluence. The medium was then replaced with a new medium containing vehicle (DMSO) or Necrox-5 (10 *μ*mol/l, Enzo Life Science) and incubated overnight at 37 °C.^22^

### Cytosolic or nuclear protein extractions

Lungs were removed and homogenized in two volumes of buffer A (50 mmol/l Tris-HCl, pH 7.5, 1 mmol/l EDTA, 10% glycerol, 0.5 mmol/l DTT, 5 mmol/l MgCl_2_ and 1 mmol/l PMSF) containing protease inhibitor cocktails. The homogenates were centrifuged at 1000 × *g* for 15 min at 4 °C. The supernatants collected were incubated on ice for 10 min and centrifuged at 100 000 × *g* for 1 h at 4 °C to obtain cytosolic proteins for analysis of NF-кB p65. The pellets were washed twice in buffer A, resuspended in buffer B (1.3 mol/l sucrose, 1.0 mmol/l MgCl_2_, and 10 mmol/l potassium phosphate buffer, pH 6.8) and then pelleted at 1000 × *g* for 15 min. The pellets were suspended in buffer B with a final sucrose concentration of 2.2 mol/l and centrifuged at 100 000 × *g* for 1 h. The resulting pellets were washed once with a solution containing 0.25 mol/l sucrose, 0.5 mmol/l MgCl_2_, and 20 mmol/l Tris-HCl, pH 7.2, and centrifuged at 1000 × *g* for 10 min. The pellets were solubilized with a solution containing 50 mmol/l Tris-HCl (pH 7.2), 0.3 mol/l sucrose, 150 mmol/l NaCl, 2 mmol/l EDTA, 20% glycerol, 2% Triton X-100, 2 mmol/l PMSF and protease inhibitor cocktails. The mixture was kept on ice for 1 h with gentle stirring and centrifuged at 12 000 × *g* for 30 min. The resulting supernatant was used as soluble nuclear proteins for analysis of NF-кB p65. The protein levels were analyzed by western blotting using anti-NF-кB p65 antibody (Upstate Biotech, Lake Placid, NY, USA) as described above.

### Determination of airway responsiveness to methacholine

Both noninvasive and invasive measurements of airway responsiveness were used in this study. For noninvasive measurement, airway responsiveness was assessed in unrestrained and conscious mice at 48 h after the last challenge with OVA, as described previously.^53^ Mice were placed in a barometric plethysmographic chamber (All Medicus Co., Seoul, Korea) and baseline readings were taken and averaged for 3 min. Aerosolized methacholine in increasing concentrations (2.5–50 mg/ml) were nebulized through an inlet of the main chamber for 3 min. Readings were taken and averaged for 3 min after each nebulization. Penh, calculated as (expiratory time/relaxation time–1) × (peak expiratory flow/peak inspiratory flow), according to the manufacturers' protocol, is a dimensionless value that represents a function of the proportion of maximal expiratory to maximal inspiratory box pressure signals and a function of the timing of expiration. Penh was used as a measure of airway resistance to methacholine. Results were expressed as the percentage increase of Penh following challenge with each concentration of methacholine, where the baseline Penh (after saline challenge) was expressed as 100%. Penh values averaged for 3 min after each nebulization were evaluated.

Invasive measurement of airway responsiveness was performed as described elsewhere.^54^ Anesthesia was achieved through intraperitoneal injection of 45 mg/kg body weight of sodium pentobarbital. The trachea was then exposed through midcervical incision, tracheostomized and an 18-gauge metal needle was inserted. Mice were connected to a computer-controlled small animal ventilator (flexiVent, SCIREQ, Montreal, Canada). The mouse was quasi-sinusoidally ventilated with nominal tidal volume of 10 ml/kg body weight at a frequency of 150 breaths/min and a positive end-expiratory pressure of 2 cm H_2_O to achieve a mean lung volume close to that during spontaneous breathing. This was achieved by connecting the expiratory port of the ventilator to water column. Methacholine aerosol was generated with an in-line nebulizer and administered directly through the ventilator. To determine the differences in airway response to methacholine, each mouse was challenged with methacholine aerosol in increasing concentrations (5.0–50 mg/ml in saline). After each methacholine challenge, the data of calculated R_rs_ were continuously collected. Maximum values of R_rs_ were selected to express changes in airway function, which was represented as a percentage change from the baseline after saline aerosol.

### Statistics

We used SPSS statistical software (version 18.0, SPSS, Chicago, IL, USA). Data are expressed as mean±S.E.M. Statistical comparisons were performed using one-way ANOVA followed by the Scheffe's test. A value of *P*<0.05 was considered statistically significant.

## Abbreviations

BAL: bronchoalveolar lavage
DAMPs: damage-associated molecular pattern molecules
DCF-DA: *2',7'*-dichlorofluorescein-diacetate
HDM: house dust mite
IL-1R: IL-1 receptor
KO: knock-out
LPS: lipopolysaccharides
MPO: myeloperoxidase
mtDNA: mitochondrial DNA
NADPH: nicotinamide adenine dinucleotide phosphate
NF-κB: nuclear factor-κB
NLR: nucleotide-binding domain, leucine-rich repeat-containing protein
OVA: ovalbumin
OVA_LPS_-OVA mice: mice sensitized with OVA and LPS and challenged with OVA
PAMPs: pathogen-associated molecular pattern molecules
Penh: enhanced pause
PRRs: pattern recognition receptors
ROS: reactive oxygen species
R_rs_: respiratory system resistance
SAL-SAL mice: saline-sensitized and -challenged mice
TLR: toll-like receptor
