# Supplementary material for: NLRP3 inflammasome activation by mitochondrial ROS in bronchial epithelial cells is required for allergic inflammation
Source: Cell Death Dis. 2014 Oct 30;5(10):e1498–. doi: 10.1038/cddis.2014.460 (PMC4237270; doi:10.1038/cddis.2014.460)
Supplement: Supplementary Information [file cddis2014460x1.doc]

### Responses to the Editor’s and Reviewers' Comments

We appreciate very much the editor and the reviewers for the constructive comments. We also thank the editor and the reviewers for the effort and time put into the review of the manuscript. Each comment has been carefully considered point by point and responded. Responses to the reviewers and changes in the revised manuscript are as follows.

**Manuscript number: CDDIS-14-0467-T**

**Title: NLRP3 inflammasome activation by mitochondrial ROS in bronchial epithelial cells is required for allergic inflammation**

**COMMENTS FROM REFEREE #1:** Thank you for your thoughtful and thorough review of our revised manuscript.

**General comments**

**COMMENTS FROM REFEREE #1:**

**In the manuscript "NLRP3 inflammasome activation by mitochondrial ROS in bronchial epithelial cells is required for allergic inflammation" by Kim et al. the role of mitochondrial ROS in allergic airway inflammation is addressed. The authors provide interesting data demonstrating that airway inflammation is accompanied by increased levels of mitochondrial ROS, which in turn activates NLRP3 inflammasome and modulate downstream inflammation. The number of papers has demonstrated that indeed factors associated with mitochondrial damage and oxidative stress could sustain NLRP3 activation (Heid et al, 2013 J immunol, Lyer SS et al, 2013 Immunity).
 This study is investigating a rapidly growing field, which aims to assess the role of mitochondrial damage in the different pathological conditions. It has been demonstrated that mitochondria-targeted antioxidant MitoQ ameliorates experimental mouse colitis by suppressing NLRP3 inflammasome-mediated inflammatory cytokines. Therefore, the experimental intervention used by the authors could be highly relevant in other models of airway inflammation including chronic obstructive pulmonary disease, ozone induced inflammation, fine particles induced airway inflammation etc. The manuscript represents novel ideas, well written.**

**Major comments:**

**The authors should assess allergic asthma phenotype in mice in a more detailed way; therefore the following parameters need to be analyzed:

1) Since in the model of allergic asthma used by the authors only very modest eosinophils attraction in the BAL could be found i.e. (1x10*4/ml), while very high numbers of neutrophils and lymphocytes were observed after OVALPS-OVA treatment. I would recommend to use alternative murine models of asthma, since it has been shown that the experimental models in the presence/absence of adjuvants could be controlled by different mechanisms (Willart et al, JEM 2012, Krysko et al, Allergy 2013). Murine model of house dust mite induced asthma model could be used as an additional experimental model.**

**Response:** We appreciate your comments very much. As you recommended, we have performed the additional experiments using a murine model of house dust mite (HDM)-induced asthma. For this animal model, mice were sensitized intratracheally with HDM extract (100 μg, Demarophagoides pteronyssius, GREER laboratories, Lenoir, NC) on days 1 and 7. On day 14 after the initial sensitization, the mice were challenged intratracheally with 100 μg of HDM extract in saline (or with saline as a control). HDM-instilled mice showed the typical allergic asthmatic features including the increased levels of airway inflammatory cells, peribronchial and perivascular inflammation, and airway hyperresponsiveness. In addition, we also found significant increases in the generation of mitochondrial reactive oxygen species (ROS) in BAL cells and the expression of NLRP3, IL-1β, and caspase-1 in lung tissues of HDM-instilled mice (Fig. 1). These all elevated parameters were dramatically reduced by the administration of NecroX-5. These findings suggest that mitochondrial ROS can contribute to the pathogenesis of allergic airway inflammation induced by a variety of allergens including OVA and HDM. These results have been incorporated into the Result section and Methods section of our revised manuscript [marked] (page 14, line 7-page 15, line 10, page 25, line 14-line 18, and revised figure 6).

**NecroX-5 reduces allergic airway inflammation, bronchial hyperresponsiveness, the activation of NLRP3 inflammasome, and the generation of mitochondrial ROS in lung of HDM-instilled mice.** Numbers of total cells, eosinophils, neutrophils, and lymphocytes in BAL fluids of HDM-instilled mice were increased significantly compared to the numbers of saline-instilled mice administered drug vehicle (SV). The increase in numbers of these cells, especially eosinophils, in BAL fluids from HDM-instilled mice was significantly reduced by administration of NecroX-5 (Fig. 1a). Histological assessment showed that numerous inflammatory cells infiltrated into the bronchioles and perivascular regions in the lung of HDM-instilled mice (Fig. 1c) compared to the SV mice (Fig. 1b). The HDM-instilled mice treated with NecroX-5 showed marked reduction in the infiltration of inflammatory cells in the peribronchiolar and perivascular regions of the lung (Fig. 1d,e). Airway responsiveness was assessed by the measurement of respiratory system resistance (R_rs_). In HDM-instilled mice, the methacholine dose-response curve of R_rs_ shifted to the left compared to that of SV mice (Fig. 1f). Administration of NecroX-5 reduced substantially the R_rs_ observed at 10 to 50 mg/ml of methacholine in HDM-instilled mice. Western blot analysis revealed that protein levels of NLRP3, caspase-1, and IL-1β in lung tissues of HDM-instilled mice were greatly increased at 48 hours after the last challenge compared to the levels in SV mice (Fig. 1g-l). The administration of NecroX-5 substantially decreased the expression of these proteins in lung tissues of HDM-instilled mice. Intensity of fluorescence was significantly higher in BAL cells (Fig. 1m) from HDM-stilled mice than that from control mice. The enhancement of fluorescence intensity in BAL cells from HDM-instilled mice was inhibited by treatment with NecroX-5.

**
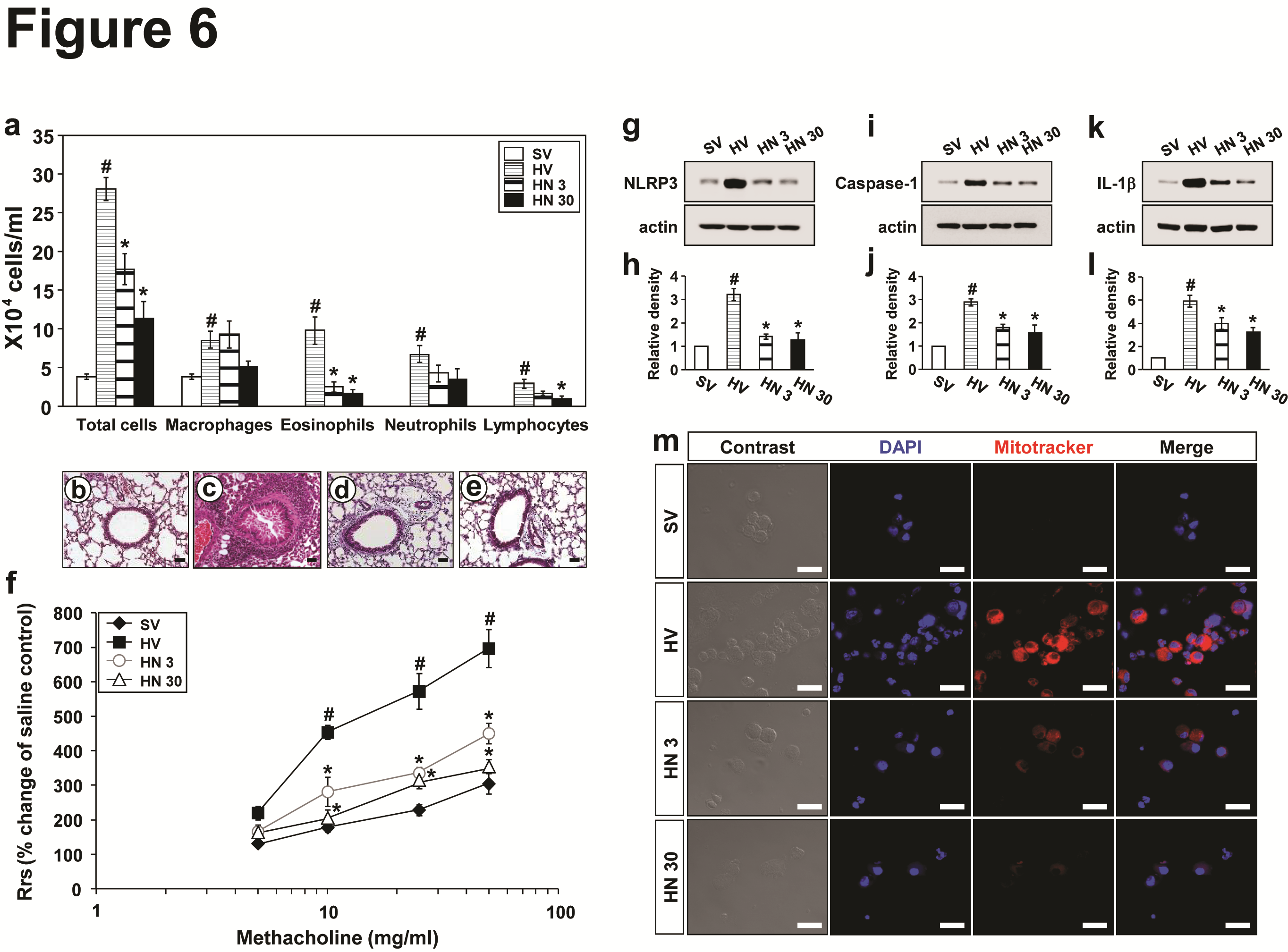
**

**Figure 1. Effects of NecroX-5 on total and differential cell counts in BAL fluids, histological changes, airway hyperresponsiveness, the protein levels of the NLRP3, IL-1β, and caspase-1, and the generation of mitochondrial ROS in the lung of HDM-instilled mice.** All parameters were measured at 48 hours after the last challenge in saline-instilled mice administered drug vehicle (SV), HDM-instilled mice administered drug vehicle (HV), HDM-instilled mice administered 3 mg/kg of NecroX-5 (HN 3) and HDM-instilled mice administered 30 mg/kg of NecroX-5 (HN 30). (**a**) Cellular changes in BAL fluids of HDM-instilled mice. Bars represent mean ± SEM from 6 mice per group. (**b-e)** Representative H&E stained sections of the lungs isolated from SV (**b**), HV (**c**), HN 3 (**d**), and HN 30 (**e**). Bars indicate scale of 20 μm. (**f**) Airway responsiveness in HDM-instilled mice was assessed by invasive measurement (R_rs_). (**g-l**) Representative Western blots for NLRP3 (**g**), caspase-1 (**i**), and IL-1β (**k**) in lung tissues and densitometric analysis. Bars represent mean ± SEM from 6 mice per group. (**m**) Representative confocal laser immunofluorescence photomicrography of BAL cells showed the localization of mitochondrial ROS (red fluorescence views) in the cells. The blue fluorescent DAPI stain was used for nuclear localization. The left panels and the right panels presented phase contrast views and the merger views, respectively. Bars indicate scale of 20 μm. ^#^*P* < 0.05 versus SV; ^*^*P* < 0.05 versus HV.

**2) OVA specific IgE, OVA-IgG1, IgG2 levels in serum need to be analyzed.**

**Response:** Thank you very much for your comment. As you commented, we measured the levels of OVA-specific IgE, OVA-specific IgG1, and OVA-specific IgG2 in serum of OVA_LPS_-OVA mice. Significant increases in OVA-specific IgE, IgG1, and IgG2 levels in the serum of OVA_LPS_-OVA mice were observed compared to the levels in the control mice (Fig. 2). Administration of NecroX-5 substantially lowered the serum level of OVA-specific IgE compared to the level of OVA_LPS_-OVA mice treated with drug vehicle only. In addition, the serum level of OVA-specific IgG1, the Th2-type immunoglobulin was decreased by treatment with NecroX-5, while the level of OVA-specific IgG2a was not affected by NecroX-5 although the level of IgG2a showed the increased tendency on the treatment with 30 mg/kg of NecroX-5 in OVA_LPS_-OVA mice. These results have been added to Results section and Methods section of our revised manuscript [marked] (page 12, line 18-page 13, line 8, page 32, line 12-line 15, and revised figure 4i-k).

**Effects of NecroX-5 on the serum level of OVA-specific IgE, -IgG1, and –IgG2a in OVA_LPS_-OVA mice.** Enzyme immunoassays showed the significant increases in OVA-specific IgE, IgG1, and IgG2 levels in the serum of OVA_LPS_-OVA mice compared to the levels in the control mice (Fig. 2). Administration of NecroX-5 substantially lowered the serum level of OVA-specific IgE compared to the levels of OVA_LPS_-OVA mice treated with drug vehicle only. In addition, the serum level of OVA-specific IgG1, the Th2-type immunoglobulin was decreased by treatment with NecroX-5, while the level of OVA-specific IgG2a was not affected by NecroX-5 although the level of IgG2a showed the increased tendency on the treatment with 30 mg/kg of NecroX-5 in OVA_LPS_-OVA mice.

**
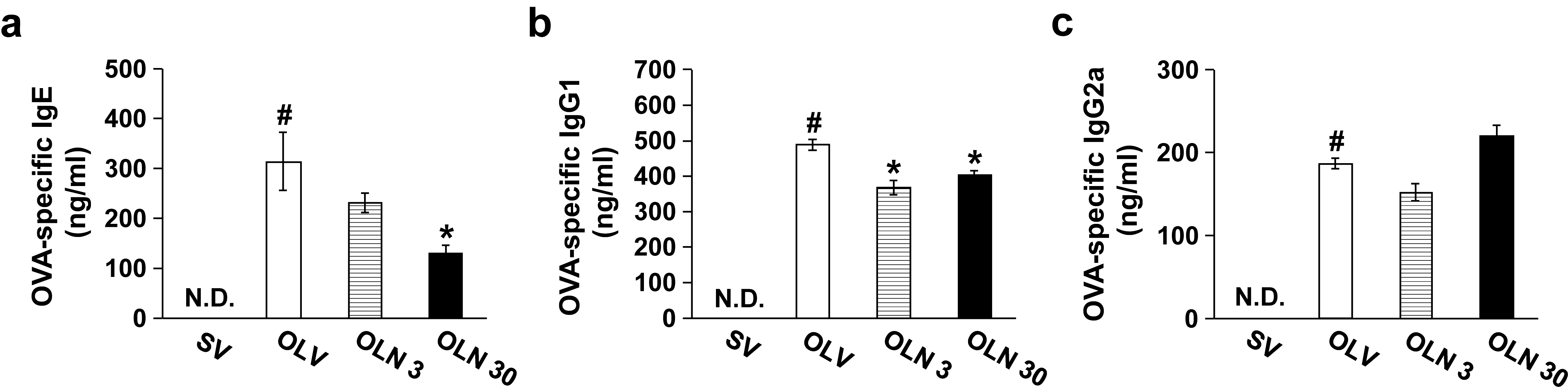
**

**Figure 2. The serum level of OVA-specific IgE, -IgG1, and –IgG2a in OVA_LPS_-OVA mice.** Sampling was performed at 48 hours after the last challenge in SAL-SAL mice administered with drug vehicle (SV), OVA_LPS_-OVA mice administered with drug vehicle (OLV), OVA_LPS_-OVA mice administered with 3 mg/kg NecroX-5 (OLN 3), or OVA_LPS_-OVA mice administered with 30 mg/kg NecroX-5 (OLN 30). Changes of the serum levels of OVA-specific IgE (**a**), OVA-specific IgG1 (**b**), and OVA-specific IgG2a (**c**). Data represent mean ± SEM from 6 mice per group. N.D., Not detected, ^#^*P* < 0.05 versus SV; ^*^*P* < 0.05 versus OLV.

**3) Were the levels of mitDNA changed in the OVALPS-OVA? Does Necrox-5 treatment influence the levels of mitDNA?**

**Response:** We appreciate your comments. To evaluate whether mitochondrial DNA (mtDNA) are changed or damaged in OVA_LPS_-OVA mice, we have performed long PCR experiments to detect mtDNA lesions hampering polymerase progress and altering replication^1^. In addition, we have measured the absolute levels of mtDNA in lung tissues of mice. First, to perform long PCR experiments, total DNA was extracted from lung tissue samples using DNAZOL reagent according to manufacturer’s protocol (Gibco/Life Technologies, Carlsbad, CA). This long PCR technique is based on the amplification of a long (8642-bp) and a short (316-bp) mtDNA fragment. The primers used were as follows: short mtDNA fragment, sense: 5’-CGACAGCTAAGACCCAAACTGGG-3’, antisense: 5’-CCCATTT CTTCCCATTTCATTGGC-3’, and long mtDNA fragment, sense: 5’-TACTAGTCCGCGAGCCTTCAAAGC-3’, antisense: 5’-GGGTGATCTTTGTTTGC GGGT-3’. PCR reactions were performed in a thermocycler (GeneAmps PCR System 2400, Applied Biosystems, Foster City, CA). The thermocycler profile for short mtDNA fragment include initial denaturation at 94°C for 2 minutes, denaturation at 95°C for 45 seconds, annealing at 61°C for 10 seconds and extension at 68°C for 8 minutes, and final extension at 68°C for 7 minutes, while for long mtDNA fragment, the profile is follows: initial denaturation at 75°C for 2 minutes and 94°C and 1 minute, denaturation at 94°C for 15 seconds, annealing at 59°C for 30 seconds and extension at 65°C for 12 minutes, and final extension at 72°C for 10 minutes. The amplified PCR products were electrophoresed using 1.6% agarose gels stained with ethidium bromide. DNA bands were visualized using Fuji film LAS-3000 (Fuji film, Tokyo, Japan) under ultraviolet transillumination.

To measure the content of mtDNA, mitochondria were isolated from lung tissues of mice by differential centrifugation. mtDNA was isolated using Mitochondrial DNA isolation kit (Biovision Inc., Mountain view, CA) and dissolved in Tris-EDTA [10mmol/L Tris∙HCl (pH 8.0) and 1 mmol/L EDTA] buffer. Samples were quantified to a final concentration of 1ng/μL. Protein levels were determined using Bradford reagent (Bio-Rad Laboratories, Hercules, CA). The levels of mtDNA were presented as ng/1 μg of protein/1 g of lung tissues. These results have been added to Results section and Methods section of our revised manuscript [marked] (page 8, line 14-page 9, line 12, page 28, line 4-page 29, line 9, and revised figure 2).

**Effects of NecroX-5 on the mtDNA levels and integrity in lung tissues of OVA_LPS_-OVA mice.** The amplification of mtDNA showed that the level of long mtDNA fragment was decreased in the lung tissues of OVA_LPS_-OVA mice compare to the level of SV mice, while short mtDNA fragment was not changed significantly in all group tested (Fig.3a). The decrease in amplified products of long mtDNA fragment was restored substantially by treatment with NecroX-5. Consistent with these results, the ratio of long mtDNA fragment/short mtDNA fragment as a parameter of the integrity of mtDNA was significantly decreased in lung tissues of OVA_LPS_-OVA mice compared to the levels of SV mice. Interestingly, the decreased ratio was substantially restored by the treatment with NecroX-5 (Fig. 3b). In addition, mtDNA content was also reduced in lung tissues of OVA_LPS_-OVA mice compare to that in SV mice. The decreased level of mtDNA was markedly restored to the similar levels of SV mice by treatment with NecroX-5 (Fig. 3c). These results suggest that mitochondrial ROS can affect the integrity and content of mtDNA in lung tissues of an asthmatic murine model.


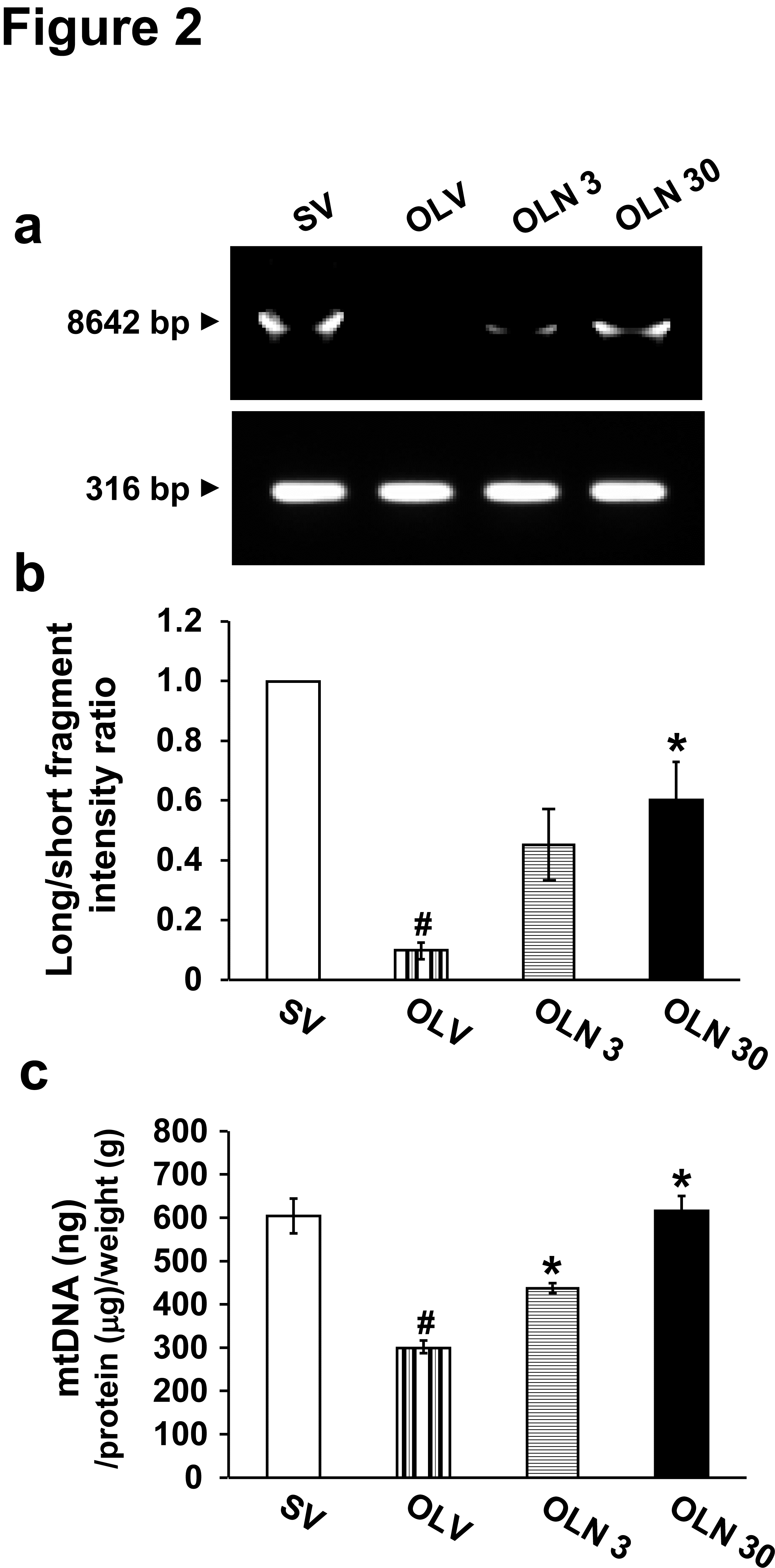


**Figure 3. Effects of NecroX-5 on the integrity and content of mtDNA in lung tissues of OVA_LPS_-OVA mice.** Sampling was performed at 48 hours after the last challenge in SAL-SAL mice administered drug vehicle (SV), OVA_LPS_-OVA mice administered drug vehicle (OLV), OVA_LPS_-OVA mice administered 3 mg/kg NecroX-5 (OLN 3), or OVA_LPS_-OVA mice administered 30 mg/kg NecroX-5 (OLN 30). (**a**) Representative PCR analyses for both a long (8642-bp) mtDNA fragment and a short (316-bp) mtDNA fragment from the lung tissues of OVA_LPS_-OVA mice. (**b**) The long fragment/short mtDNA fragment hybridization ratio in lung tissues of OVA_LPS_-OVA mice. (**c**) Changes of the content of mtDNA in the lung tissues of OVA_LPS_-OVA mice. Data represent mean ± SEM from 6 mice per group. ^#^*P* < 0.05 versus SV; ^*^*P* < 0.05 versus OLV.

**Reference**

1. Mansouri, A. *et al.* An alcoholic binge causes massive degradation of hepatic mitochondrial DNA in mice. *Gastroenterology* **117**, 181–190 (1999).

**4) The authors suggest that mitochondrial ROS play a critical role in the pathogenesis of OVALPS-OVA murine model of airway inflammation through the modulation of NLRP3 inflammasome activation. To prove this hypothesis it would be necessary to test the above mentioned murine model of OVALPS-OVA induced airway inflammation in IL-1R KO or NALP3 KO mice. Alternatively refer to already published research.**

**Response:** Thank you very much for the suggestion. As you suggested, we have performed additional experiments using IL-1R knock-out (KO) mice. Female C57BL/6 IL-1R KO mice and wild type (WT) mice, 8 to 10 weeks of age and free of murine specific pathogens, were obtained from the Jackson laboratory. (Sacramento, CA). They were housed throughout the experiments in a laminar flow cabinet, and were maintained on standard laboratory chow *ad libitum*. Under the same experimental protocol to original experiments, the IL-1R KO mice were cared and treated. OVA_LPS_-OVA IL-1R KO mice showed the significantly reduced asthmatic manifestations including the number of airway inflammatory cells, pathologic changes, and airway hyperresponsiveness compared to the levels of OVA_LPS_-OVA wild type (WT) mice (Fig. 4). In addition, the IL-1R KO mice showed the lower levels of pro-inflammatory mediators such as IL-4, IL-5, IL-13, IL-17, and KC in lung tissues after OVA challenges than the levels of OVA_LPS_-OVA WT mice (Fig. 5). These findings support our contention that mitochondrial ROS play a critical role in the pathogenesis of OVA_LPS_-OVA murine model of airway inflammation through the modulation of NLRP3 inflammasome activation. These data have been added to Results section and Methods section of our revised manuscript [marked] (page 15, line 12-page 16, line 1, page 16, line 18-page 17, line 5, page 25, line 4-line 6, and revised figure 7g-l and 8o-x).

**Changes of BAL cells, histologic features, and airway hyperresponsiveness in OVA_LPS_-OVA IL-1R KO mice.** To block the effects of IL-1β, an indicator of NLRP3 inflammasome activation, on changes in BAL cells, histologic features, and airway hyperresponsiveness, IL-1R KO mice were used. The increased numbers of BAL cells in OVA_LPS_-OVA IL-1R KO mice were substantially lower than the levels of OVA_LPS_-OVA WT mice (Fig. 4a). In addition, the OVA_LPS_-OVA IL-1R KO mice showed a marked reduction in infiltration of numerous inflammatory cells into peribronchiolar and perivascular regions on histologic examination (Fig. 4b-e). Airway responsiveness assessed by a percent increase of R_rs_ in response to increasing doses of methacholine revealed that the percent R_rs_ of OVA_LPS_-OVA IL-1R KO mice was significantly lower than that of OVA_LPS_-OVA IL-1R WT mice (Fig. 4f).


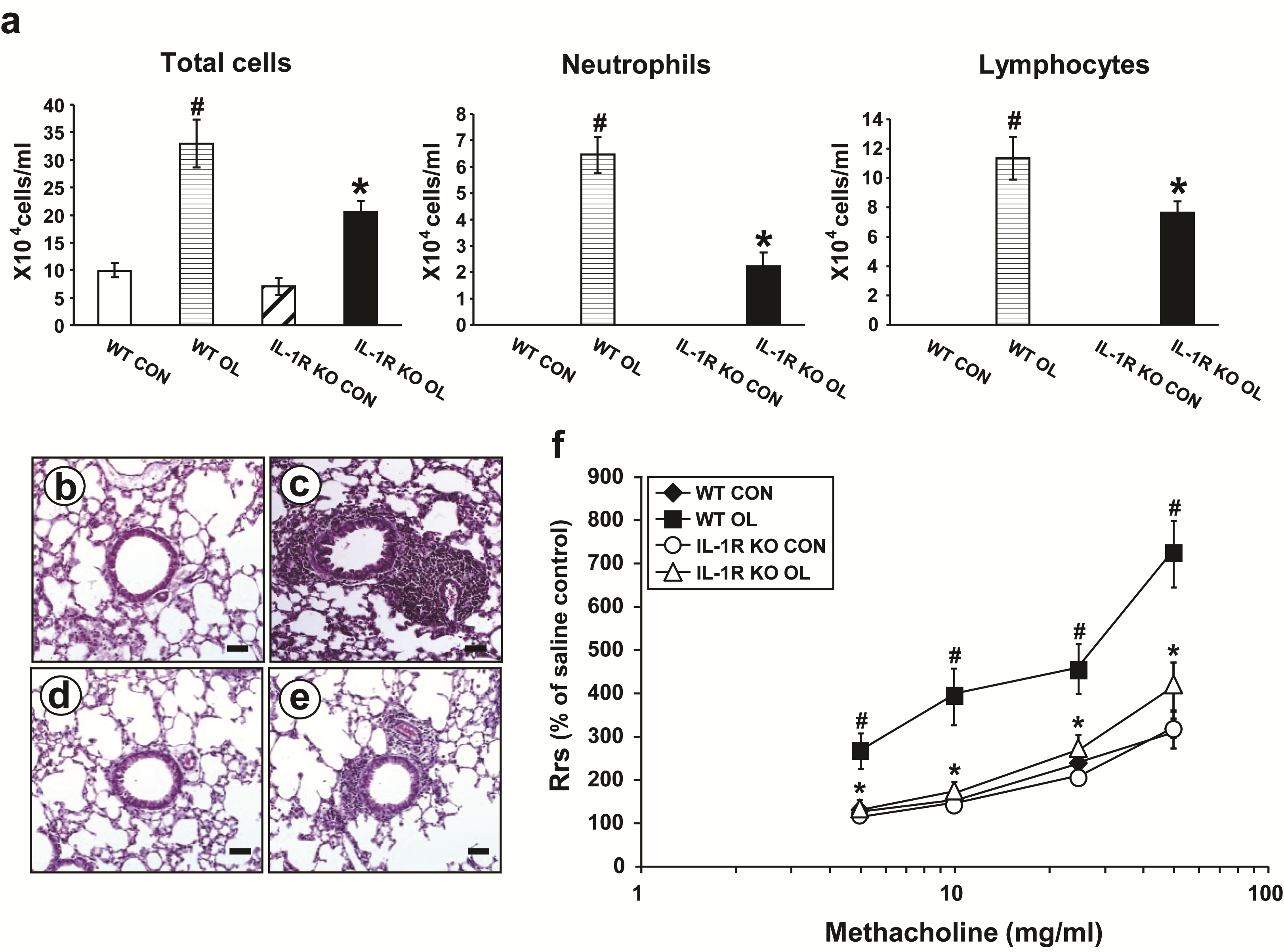


**Figure 4.** **Total and differential cell counts in BAL fluids, histological changes, and airway hyperresponsiveness of OVA_LPS_-OVA IL-1R KO mice.** All parameters were measured at 48 hours after the last challenge in SAL-SAL WT mice (WT CON), OVA_LPS_-OVA WT mice (WT OL), SAL-SAL IL-1R KO mice (IL-1R KO CON), and OVA_LPS_-OVA IL-1R KO mice (IL-1R KO OL). (**a**) Cellular changes in BAL fluids of IL-1R KO or WT mice. Bars represent mean ± SEM from 5 mice per group. (**b-e)** Representative H&E stained sections of the lungs isolated from WT CON (**b**), WT OL (**c**), IL-1R KO CON (**d**), and IL-1R KO OL (**e**). Bars indicate scale of 50 μm. **(f)** Airway responsiveness of IL-1R KO or WT mice was assessed by the invasive measurement (R_rs_). Bars represent mean ± SEM from 5 mice per group. ^#^*P* < 0.05 versus WT CON; ^*^*P* < 0.05 versus WT OL.

**Changes in levels of inflammatory cytokines in the lung tissues of OVA_LPS_-OVA IL-1R KO mice.** Western blot analyses showed that protein levels of IL-4, IL-5, IL-13, IL-17, and KC protein in lung tissues of OVA_LPS_-OVA IL-1R KO mice were markedly lower than the levels in OVA_LPS_-OVA WT mice (Fig. 5).

**
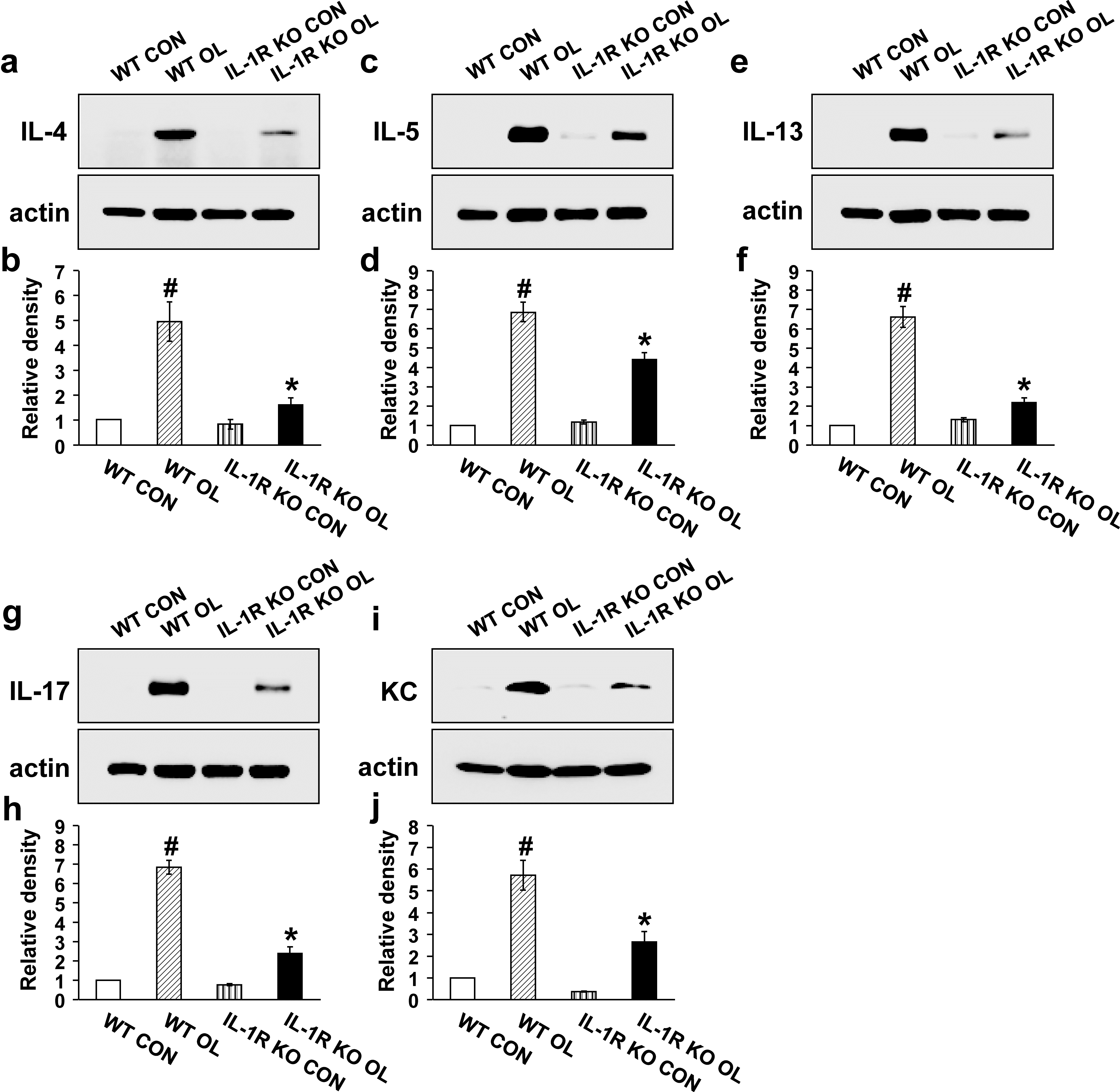
**

**Figure 5. Protein levels of various inflammatory mediators in lung tissues of OVA_LPS_-OVA IL-1R KO mice.** Representative Western blots of IL-4 (a), IL-5 (c), IL-13 (e), IL-17 (g), and KC (i) in lung tissues. Densitometric analysis is presented as the relative ratio of each protein to actin. The relative ratio of each protein in the lung tissues of WT CON is arbitrarily presented as 1. Bars represent mean ± SEM from 5 mice per group. ^#^*P* < 0.05 versus WT CON; ^*^*P* < 0.05 versus WT OL.

**Minor remarks:

Fig.1 please make all abbreviations consistent. Fig. 1e and Fig. 1f mention dose of OLN as in Fig.1a and 1b. Fig.1b please provide mean intensity as in Fig.1f**

**Response:** Thank you for your comments. As you commented, we have corrected the abbreviations to be consistent and provided the mean intensity in Fig.1b.

**Fig.2 were the levels of IL-1b analyzed in BAL of asthmatics patients? Fig.2 m please specify in Fig. legend if human or murine samples were analyzed.**

**Response:** As you pointed out, we have specified the legend of Fig.2 to be clearer which figure presents the data from human or mice in our revised manuscript [marked] (page 52, line 1-3, line 7-8, and line 10)

**Since authors claim that mitochondrial ROS acts via IL-1b mediated mechanism. The above mentioned experiments need to be repeated in IL-1 KO mice.**

**Response:** We appreciate your comment very much. As you commented, we have performed the additional experiments using IL-1R KO mice as described above.

**Please correct spelling mistakes in the text.**

**Response:** We have corrected spelling mistakes in the entire text.

Yong Chul Lee, MD, Ph D

Professor

Department of Internal Medicine

Chonbuk National University Medical School

San 2-20, Geumam-dong, Deokjin-gu, Jeonju

Jeonbuk, 561-180, South Korea

Phone: 82 63 250 1664

Fax: 82 63 259 3212

E-mail: [leeyc@jbnu.ac.kr](mailto:leeyc@jbnu.ac.kr)
